# Effect of Inoculation with Arbuscular Mycorrhizal Fungi (*Rhizophagus irregularis* BGC AH01) on the Soil Bacterial Community Assembly

**DOI:** 10.3390/jof11100739

**Published:** 2025-10-15

**Authors:** Xueli Wang, Xuemin Jing, Yan Wang, Youran Ma, Xiangyang Shu, Wei Fu, Shuping Xing, Weijia Liu, Qinxin Ye, Yalan Zhu, Ping Ren, Xin Zhang, Baodong Chen, Xia Wang

**Affiliations:** 1 The Key Laboratory of Land Resources Evaluation and Monitoring in Southwest China, College of Geography and Resources, Sichuan Normal University, Chengdu 610066, China; wangxueli@stu.sicnu.edu.cn (X.W.); xyshu@sicnu.edu.cn (X.S.); zhuyalan2025@163.com (Y.Z.); renping@sicnu.edu.cn (P.R.); 2 State Key Laboratory for Ecological Security of Regions and Cities, Research Center for Eco-Environmental Sciences, Chinese Academy of Sciences, Beijing 100085, China; jxm6452@163.com (X.J.); weifu@rcees.ac.cn (W.F.); bdchen@rcees.ac.cn (B.C.); 3 Heilongjiang Provincial Key Laboratory of Ecological Restoration and Resource Utilization for Cold Region, School of Life Sciences, Heilongjiang University, Harbin 150080, China; 4 School of Chemistry and Materials Science, Sichuan Normal University, Chengdu 610066, China; wangyanwangyan@stu.sicnu.edu.cn; 5 China Aerospace Construction Group Co., Ltd., Beijing 100071, China; superyoyo9938@163.com; 6 Information Center of Ministry of Ecology and Environment, Beijing 100029, China; stellar_xsp@sina.com; 7 Institute of Agricultural Bioenvironment and Energy, Chengdu Academy of Agriculture and Forestry Sciences, Chengdu 611130, China; liuweijia27@163.com (W.L.); yeqinxin123@163.com (Q.Y.); 8 University of Chinese Academy of Sciences, Beijing 100049, China

**Keywords:** arbuscular mycorrhizal fungi, *Rhizophagus irregularis*, bacterial community assembly, deterministic processes, temporal dynamics

## Abstract

Soil bacterial communities are crucial drivers of nutrient cycling and ecosystem functioning; however, their temporal dynamics under arbuscular mycorrhizal (AM) fungi colonization remain insufficiently characterized. In this study, we used a non-destructive continuous sampling method and undertook a 90-day pot experiment to examine the process of shaping the bacterial community of hyphosphere soil. Following inoculation with AM fungi, we found an increase in the α-diversity index of the hyphosphere bacterial community. The community diversity and richness and the key bacterial taxa in the hyphosphere both gradually increased from 30 to 60 days and stabilized thereafter. Principal coordinated (PCoA) analysis and network analysis further confirmed these findings. Stabilized by 60 days post-inoculation, with deterministic processes dominating assembly in inoculated AM fungi soils, while stochastic processes prevailed in non-inoculated controls. Inoculation strengthened bacterial associations with available phosphorus, while making the key bacterial communities more responsive to multiple soil physicochemical properties (available P, CEC, N, and TOC). These findings provide critical insights into AM fungi mediation of soil microbiome dynamics, with the identified 60-day stabilization period offering a key temporal framework for understanding tripartite soil–AM fungi-bacteria interactions.

## 1. Introduction

Arbuscular mycorrhizal (AM) fungi are vital to both agricultural and natural ecosystems, forming a symbiotic relationship with over 70% of terrestrial plants [1]. The hyphal exudates stimulated bacterial development and activity and altered the nutrient turnover of soil microbes [2,3]. Soil microbes are of pivotal importance in the soil environment and ecological processes [4,5]. Their activity shows how strong and in which direction different chemical reactions are [6,7]. Decreases in microbial diversity have been shown to affect the nutrients available to and absorbed by plants [8]. The microbial change trends contribute to a more profound understanding of how bacterial communities develop in soil and how nutrient cycling processes work. The recognition that soil microorganisms can exhibit temporal variation is imperative. Thus, understanding the changes in the temporal dynamics of microbial communities influenced by AM fungi is vital for the future of sustainable agriculture.

AM fungi inoculation would impact diversity and abundance levels of bacterial communities over time [9,10]. AM fungi can supply plants with mineral nutrients, particularly phosphorus (P) and nitrogen (N), and transfer carbon produced through photosynthesis via the hyphae. In the process, they interact with the microbial community [11]. Similar to root exudates, extraradical hyphae extend into soil, secreting amino acids, amines, carbohydrates, polyols, nucleic acids, and protein/peptide signaling molecules. The AM fungi hyphal exudates could recruit the specific hyphosphere microbiome, interacting with hyphosphere microbial communities, and secreted proteins may also act as fungi effectors to influence other microbes, thereby shaping these communities and facilitating plant nutrient acquisition [12]. For instance, AM fungi hyphae have been found to synergize with phosphate-solubilizing bacteria involved in the mineralization of organic phosphates. The detection of these bacteria on the surface of AM fungal hyphae suggests they play a role in facilitating the utilization of discrete organic phosphorus sources [2,11]. AM fungi can also obtain nitrogen sources either from mycelia or from nitrogen-fixing bacteria associated with mycelial hyphae [1].

The distinct processes by which soil microbial communities assemble give rise to distinctive phylogenetic patterns and community succession, which directly influence ecosystems [13]. Consequently, seedlings from the same species may be subject to divergent microbial communities, contingent on the identity of the species of conditioning plants and the duration of soil modification. Because of the dynamic and intricate nature of the soil environment, considerable variations exist in the soil community composition among different stages [14,15]. Therefore, there is a need for further exploration of the AM fungal hyphosphere microbe roles and response patterns within the hyphosphere microbial community [16].

To understand ecosystem diversity and functioning, it is vital to comprehend the interconnections that exist between community assembly and species coexistence processes. Despite the abundance of literature addressing soil improvement and restoration processes involving AM fungi, research has focused primarily on understanding their specificities and functionalities rather than on establishing bacterial communities and their characteristics. The microbial communities associated with hyphae exhibit dynamic patterns closely linked to the plant’s developmental phase [17], while the dynamics of hyphosphere microbes during plant growth are not well understood. Hence, it can be hypothesized that bacterial communities present in the hyphosphere networks of AM fungi are subject to alterations that exhibit regularity and that the community construction process is dominated by a deterministic process and is concomitant with the physicochemical characteristics of the soil.

In this experimental study, a non-destructive continuous sampling method was employed, and a 90-day pot experiment was conducted with the aim of investigating the process of shaping microbiomes in hyphosphere soil. We conducted greenhouse pot experiments using maize [18] inoculated with/without the AM fungi *Rhizophagus irregularis* (+M/−M) and grown in sterilized soils. Here, we sought to profile the hyphosphere microbial communities during the growth phase of maize, via periodic sampling at 30-day, 60-day, and 90-day intervals. This study aimed to (i) reveal the temporal dynamics of the bacterial community and of the key bacteria as the AM fungal hyphosphere develops and (ii) analyze the bacterial community assembly process and key influencing soil factors.

## 2. Materials and Methods

### 2.1. Selected Strains

*Rhizophagus irregularis* has been shown to enhance host plant growth by regulating key genes in the mycorrhizal nitrogen transport pathway, including satureja, soybean, cannabis, and basil. This, in turn, has been demonstrated to improve nutrient supply and antioxidant capacity [19,20,21]. The synergistic interaction between *Rhizophagus irregularis* and plant-associated soil microorganisms has been shown to significantly increase nitrogen uptake in host plants [22,23,24]. *Rhizophagus irregularis* Schenck & Smith BGC AH01 was provided by the Institute of Plant Nutrition and Resources of the Beijing Academy of Agriculture and Forestry Sciences. The inoculum consisted of a mixture comprising root segments, mycelium, and spores, along with sand, with a spore count of approximately 150 per gram.

### 2.2. Experimental Setup

The soil used in the experiment was obtained from the Field Experiment and Demonstration Base of the Research Center for Eco-Environmental Sciences, Chinese Academy of Sciences, which is located in Tangjiapu Village, Yanqing District, Beijing, China (40°29′ N, 115°59′ E). The fundamental physicochemical properties of the soil were determined as follows: pH 8.23; organic matter content, 19.5 g/kg; total nitrogen content, 1.02 g/kg; total phosphorus content, 0.70 g/kg; and available phosphorus content, 18.71 mg/kg. After natural air-drying, a sieving process was conducted on the soil using a 2 mm mesh, followed by a sterilization step through gamma radiation for 48 h (25 kGy, 10 MeV electron beam). Prior to use, base fertilizers were applied at rates of 30 mg/kg P, 90 mg/kg N, and 120 mg/kg K.

The bacterial communities attached to the nylon mesh in the sampler in the hyphal chamber were compared in this study with the non-inoculated and the inoculated treatments with AM fungi, as opposed to the soil attached to the hyphae. AM fungi hyphae are so tiny that separating soil samples from their surfaces is difficult. Consequently, bacteria that exhibited a high degree of colonization along the hyphal surface were utilized to ascertain both the composition and the status of this bacterial community. This is reasonable because the bacterial collective residing on a hyphal surface is considered a constituent of a broader bacterial collective within the hyphosphere.

**Figure 1:**
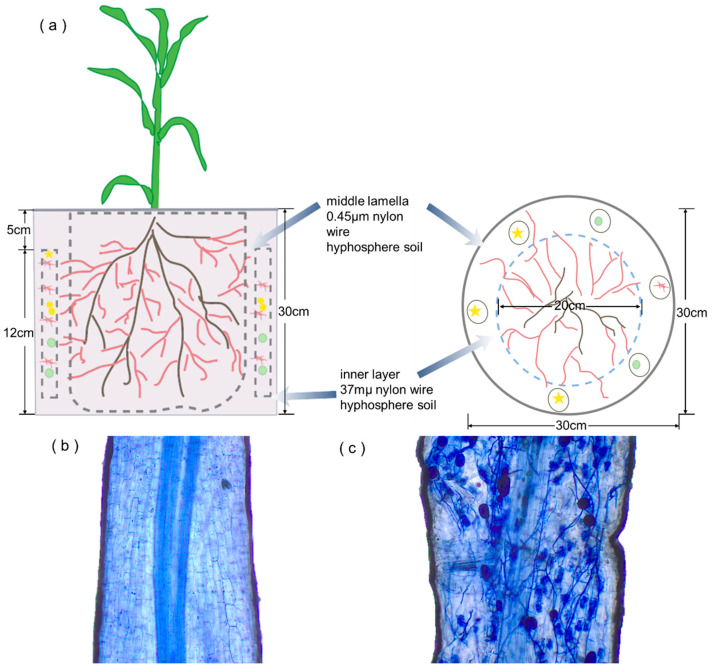
(**a**) Schematic drawing of the three-compartment cultivation system. (**b**,**c**) The AM fungi mycorrhizal infection status for non-inoculated and inoculated AM fungi treatment.

Microcosms with a diameter of 25 cm and three chambers were constructed: a root chamber (RC) for plant growth, a buffer chamber (BC), and a hyphal chamber (HC) for hyphal growth (Figure 1a). The diameter of the basin is 25 cm, and the diameters of the inner nylon meshes are 15 cm and 20 cm. A 37 μm mesh separated the three chambers. The quantity of soil added was as follows: the RC had a mass of 7 kg, the HC had a mass of 1.6 kg, and the BC had a mass of 1.6 kg. In each hyphal chamber, there was an elliptical perforated sampler with a 0.45-micron nylon mesh inside the sampler, and six were placed in each pot.

The experiment included two groups: (1) inoculated AM fungi treatment (+M) and (2) non-inoculated AM fungi treatment (–M). One maize plant was planted in each compartment of the cultivation system, which was designed with six replications of the two treatments. The maize seeds were sown on 28 August 2023 and subsequently harvested on 28 November 2023. At the point of the vegetative stage, soil samples were obtained from each treatment on 28 September (A), 28 October (B), and 28 November (C), concurrent with the performance of routine management.

### 2.3. Sample Collection

For each growth stage, we collected six soil samples from an elliptical perforated sampler with a 0.45-micron nylon mesh. Prior to being transported to the designated laboratory, the samples were placed within sterile plastic bags. The soils utilized for edaphic properties analyses were collected from the 0.45-micron nylon mesh inside the sampler by means of manual shaking. The soils that exhibited a strong adherence to the nylon mesh were subjected to a high-speed centrifugal separation process, utilizing a phosphoric acid buffer as a stabilizing agent for subsequent microbiological assays.

Subsequent to the conclusion of the experiment, extraction of the plants was carried out from the cultivation setup. The shoots and roots were then collected via different methods. Some samples were stored in a refrigerator or a freezer to analyze their physical properties, chemical composition, and bacterial sequences. After the biomass was determined, the plant roots were subjected to a treatment at 105 °C for 30 min, after which they were dried at 65 °C. During this process, the soil present in the HC, which had undergone removal of around 7 cm of topsoil, and the soil surrounding the membrane within the device were designated the hyphal soil. The soil samples were obtained via sterile sampling bags, which were meticulously marked, sealed, and stored promptly in a sampling box at a low temperature.

### 2.4. Sample Analysis

#### 2.4.1. Mycorrhizal Colonization and Microelement Concentrations

To estimate mycorrhizal colonization, 0.5 g of root was cut into segments 1 cm long and cleaned using 10% KOH, followed by a softening step with 2% HCl and, finally, a staining procedure with 0.05% trypan blue in lactic acid (Shanghai Aladdin Biochemical Technology Co., Ltd., Shanghai, China). Thirty root segments, stained for observation, were placed on microscope slides and observed under 100× magnification. The Mycocalc v0.4 software was used to calculate the mycorrhizal colonization rate (M%) and arbuscule abundance (A%) [19].

The sample was ground and then subjected to digestion with 10 mL of high-purity nitric acid at a temperature of 120 °C, via an open block digestion system (Mars 6, CEM Corporation, Matthews, NC, USA). The concentrations of phosphorus (P), potassium (K), chromium (Cr), calcium (Ca), and magnesium (Mg) were analyzed via inductively coupled plasma optical emission spectroscopy (ICP-OES; Optima 7000 DV, PerkinElmer, Waltham, MA, USA) [25].

#### 2.4.2. Determination of Soil Physicochemical Properties

In this study, the soil cation exchange capacity (CEC) was determined via the extraction via the cobalt hexaammine trichloride-spectrophotometric method. The pH of the soil was measured via the glass electrode technique [26,27]. The available phosphorus (AP) content was measured via a combination of a solution of sodium bicarbonate and the molybdenum-antimony colorimetric method [28]. Overall, samples from the experiment were collected across two distinct treatments, encompassing three stages of plant growth, with four replicates of each treatment.

#### 2.4.3. Soil Bacterial Community

The soil bacterial communities under scrutiny were examined by means of high-throughput amplicon sequencing [29]. The Amplicon Sequence Variant (ASV) table was rarefied on the basis of the lowest number of reads among all the samples, with a view to estimating the alpha diversity or beta diversity. High-throughput sequencing analysis of the bacterial rRNA genes was conducted by Biomarker Technologies in Beijing, China, using the Illumina HiSeq 2500 platform.

### 2.5. Statistical Analysis

To estimate alpha diversity, the Richness, Shannon, and Chao1 indices were used. Nonmetric multidimensional scaling analyses based on the Bray–Curtis distance were utilized to illustrate alterations in community composition that were observed between the various treatments. The corrplot package was used to analyze the correlations of the soil bacterial communities with soil physicochemical properties. One-way analysis of variance was employed, with a Tukey post hoc test subsequently used to investigate any observed differences between the groups (significance level 0.05).

## 3. Results

### 3.1. Effects of AM Fungi Inoculation on Crop Growth and Soil Physicochemical Properties

The maize established good symbiosis with AM fungi, with colonization rates of 3% in the non-inoculated plants and 85.1% in the inoculated ones (Table S1). Arbuscules, hyphae, and vesicles were identified in the root systems of plants inoculated with AM fungi (Figure 1b,c). There was a significant increase in maize biomass due to mycorrhizal colonization (*p* < 0.05, Table 1). The shoots of maize plants inoculated and not inoculated with AM fungi weighed 13.3 g and 20.5 g per pot, respectively, and their roots weighed 3.2 g and 4.3 g per pot, respectively. After inoculation, the shoot and root dry weights increased by 1.55 and 1.32 times, respectively, compared with those in the control treatment. AM fungi treatment increases soil carbon and nitrogen levels in comparison to the control treatment. Furthermore, it has been shown that AM fungi significantly reduce soil-available P and CEC during the 90-day maize growth stage (*p* < 0.05, Tables S3 and S4).

### 3.2. Effects of AM Fungi Inoculation on the Soil Bacterial Community Composition and Diversity

**Figure 2:**
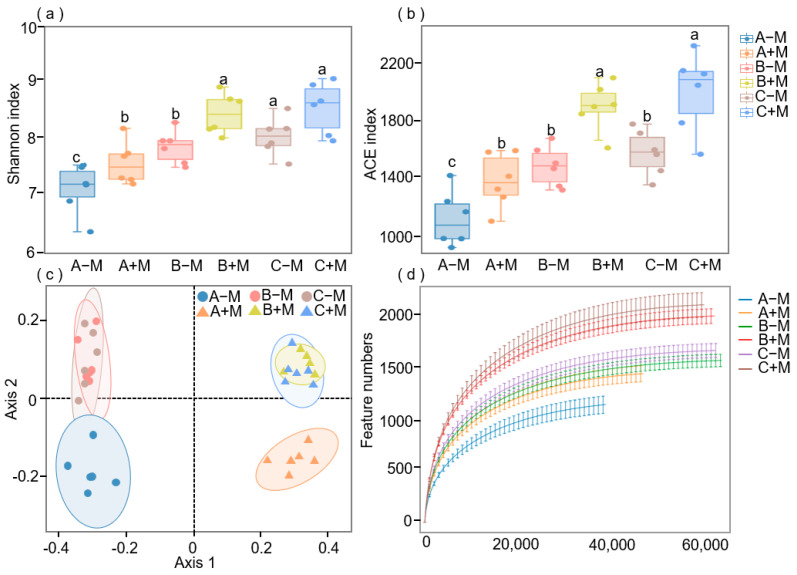
Characteristics of bacterial community diversity throughout plant growth. (**a**,**b**) Alpha diversity of the bacterial community. (**c**) PCoA based on Bray–Curtis distance matrices. (**d**) Different treatments of time series rarefaction curves. The percentage of assembly processes in the bacterial communities. A–M represent 30 days non-inoculated AM fungi treatment. B–M represent 60 days non-inoculated AM fungi treatment. C–M represent 90 days non-inoculated AM fungi treatment. A+M represent 30 days inoculated AM fungi treatment. B+M represent 60 days inoculated AM fungi treatment. C+M represent 90 days inoculated AM fungi treatment. Different lowercase letters indicate significant differences among treatments following one-way analysis of variance (*p* < 0.05).

After inoculation, the abundance of *Firmicutes*, *Gemmatimonadota*, *Actinobacteriota*, *Chloroflexi*, and *Acidobacteriota* increased gradually with plant growth, while the non-inoculated AM fungi, *Gemmatimonadota*, and *Actinobacteriota* decreased (Figure S2). Throughout the whole growth period of maize, the bacteria α-diversity showed a gradual increase from 30 to 60 days and remained stable during the period from 60 to 90 days (Figure 2a,b) (Figures S2 and S3). However, in every stage, the α-diversity values with the inoculated AM fungi are greater than those of the non-inoculated treatment. PCoA analysis revealed that AM fungi significantly altered bacterial structure. Additionally, it is noteworthy that the time of plant growth considerably changed the bacterial structure at the 30-day stage and that soil bacterial communities were obviously clustered together at 60 and 90 days under the same treatment (Figure 2c,d). 

### 3.3. Effects of AM Fungi Inoculation on the Bacterial Community Network and Assembly

The network of inoculated AM fungi had a higher node and edge number compared to the network of non-inoculated AM fungi (Figure S4 and Table S2), indicating that the former’s bacterial interactions were more complex than the latter. Throughout the whole growth period of maize, there is a marked increase in the quantity of nodes in the network between days 30 and 60, after which the rate of increase slows down (Figure S4).

**Figure 3:**
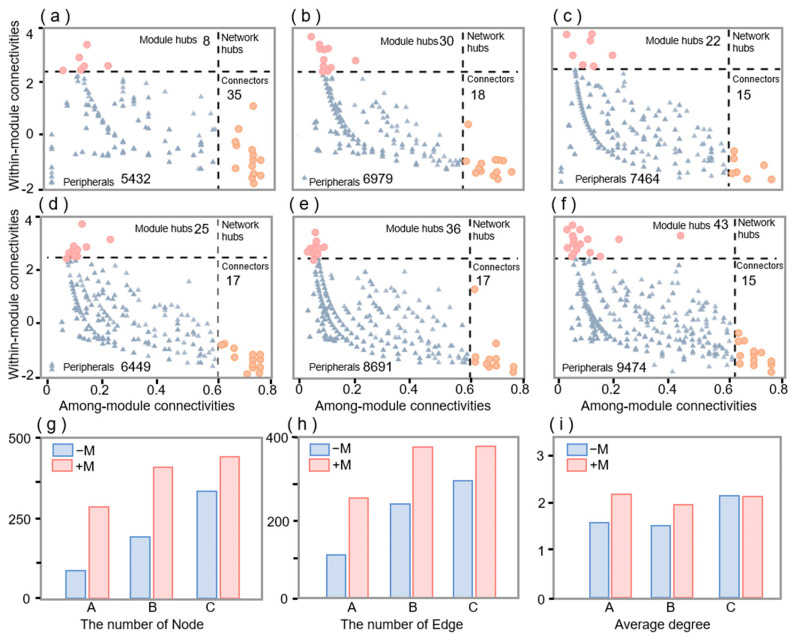
Effects of inoculated AM fungi on the keystone species of the bacterial community. (**a**,**b**), Zi-Pi plots showing the keystone species of the bacteria under different treatments. Module hubs (Zi > 2.5 and Pi < 0.62), network hubs (Zi > 2.5 and Pi > 0.62), connectors (Zi < 2.5 and Pi > 0.62), and peripherals (Zi < 2.5 and Pi < 0.62). (**a**–**c**) Non-inoculated treatment at 30, 60, and 90 days, respectively. (**d**–**f**) Inoculated AM fungi treatment at 30, 60, and 90 days, respectively. (**g**) The number of the community co-occurrence network nodes. (**h**) The number of the community co-occurrence network edges. (**i**) The average degree of the community co-occurrence network. A means the time of plant growth is 30 days; B means the time of plant growth is 60 days; C means the time of plant growth is 90 days.

Non-inoculated 43, 48, and 37 keystone species were found in the 30, 60, and 90 days, respectively (Figure 3a–c), and the keystone species of the non-inoculated treatment mainly belong to *Proteobacteria*, *Bacteroidota*, and *Firmicutes* (Tables S5–S7). While 42, 53, and 58 keystone species of inoculation were found at 30, 60, and 90 days, respectively (Figure 3d–f), this result shows that AM fungi inoculation keystone species mainly belong to *Proteobacteria*, *Firmicutes*, *Actinobacteriota*, *Bacteroidota*, and *Gemmatimonadota* (Tables S8–S10) (Figure 3a–f). At the phylum level, after 30 days of inoculation with AM fungi, there were 9 keystone species, 13 at 60 days, and 16 at 90 days. Among them, 12 keystone species were the same at 60 and 90 days (Tables S8–S10).

### 3.4. Relationships Between the Soil Bacterial Community and Soil Physicochemical Properties

**Figure 4:**
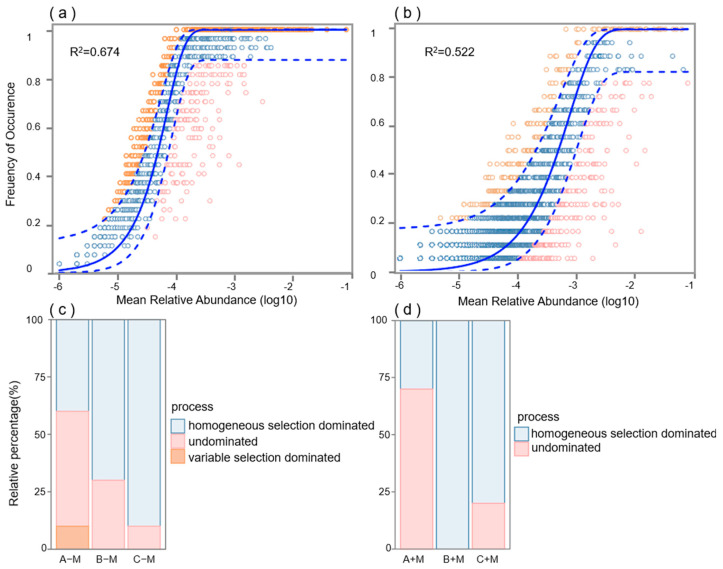
Fit of the neutral community model of the bacterial community assembly during the whole operation period: (**a**) non-inoculated and (**b**) inoculated AM fungi. The percentage of assembly processes in the bacterial communities for (**c**) non-inoculated and (**d**) inoculated AM fungi. A–M represent 30 days non-inoculated AM fungi treatment. B–M represent 60 days non-inoculated AM fungi treatment. C–M represent 90 days non-inoculated AM fungi treatment. A+M represent 30 days inoculated AM fungi treatment. B+M represent 60 days inoculated AM fungi treatment. C+M represent 90 days inoculated AM fungi treatment.

The process in the non-inoculated treatment was predominantly governed by stochastic processes. In contrast, deterministic processes were the main force influencing bacterial community assembly in the inoculated AM fungi treatment. (Figure 4b). In the non-inoculated treatment, variable selection made a substantial contribution at the 30-day stage, a feature not observed in the inoculated treatment (Figure 4c,d). At the 60-day stage, homogeneous selection became the dominant process in the inoculated treatment, whereas its role in the non-inoculated treatment was significantly smaller (approximately 30%).

**Figure 5:**
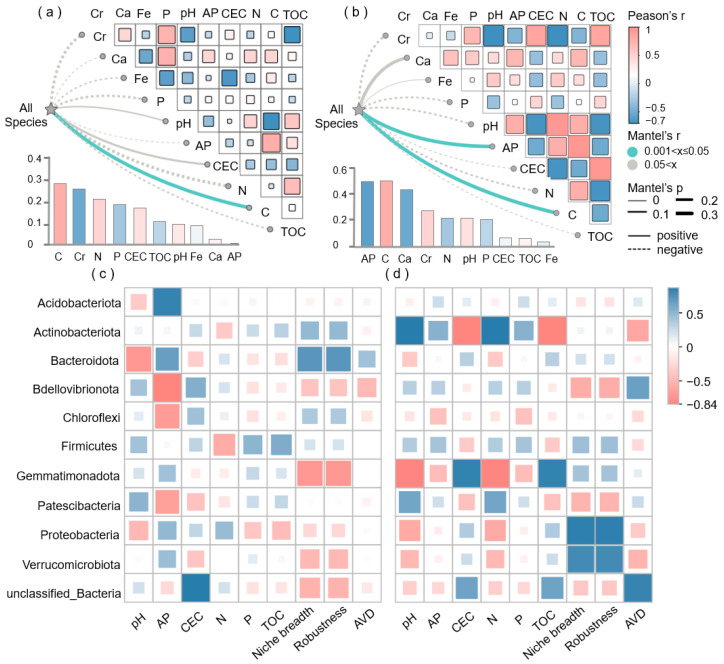
Correlation analysis of the soil parameters with the bacterial community traits. (**a**) Non-inoculated; (**b**) inoculated with AM fungi. Correlations between key hyphosphere bacteria and critical soil indicators. (**c**) Non-inoculated; (**d**) inoculated with AM fungi.

Under AM fungi inoculation, *Actinobacteriota* and *Gemmatimonadota* were significantly correlated with soil physicochemical properties and bacterial stability indices (Figure 5c,d). The key bacterial community composition of non-inoculated AM fungi was influenced predominantly by pH, available P, niche breadth, and robustness, whereas the inoculated AM fungi bacterial community composition was significantly affected by pH, available P, CEC, N, TOC, niche breadth, robustness, and AVD (average variation degree).

As shown in Figure 5a,b, the bacterial community in the non-inoculated AM fungi significantly correlated with C, Cr, and N, whereas that in the inoculated AM fungi significantly correlated with AP, C, and Ca (*p* < 0.05). The bacterial community composition in the inoculated AM fungi treatment was strongly correlated with soil trace elements (Cr, Ca, Fe, P, and Mn), while the bacterial community composition in the inoculated AM fungi treatment was strongly correlated with C, N, and TOC (Figure S6). Redundancy (RDA) analysis revealed C and N as important factors that significantly impacted bacterial communities under AM fungi inoculation (Figure S5).

## 4. Discussion

Soil microbes play a pivotal role in the function of soil ecosystems, as demonstrated by their involvement in the transformation of nutrients, the decomposition of organic matter, and the cycling of elements [30,31,32,33]. A considerable corpus of research has demonstrated the capacity of AM fungi inoculants to enhance soil fertility by means of improving the soil bacterial community, thereby promoting plant growth [34]. Furthermore, studies have shown that AM fungi (of the phylum *Glomeromycota*) can influence the abundance and diversity of microbial communities [35]. Compared with the control, inoculation with AM fungi (*Rhizophagus irregularis* BGC AH01) increased the relative abundance of bacteria in the hyphosphere (Figure S1). AM fungi increased its nutrient content and improved its condition, making it more suitable for microbial survival and reproduction [36]. The present study has shown that the presence of AM fungi mycelium can facilitate mineral weathering, enabling the acquisition of mineral nutrients [37]. The change trend of microorganisms provides a basis for understanding the formation of soil bacterial communities and nutrient accumulation. Interestingly, the community abundance reached a stable state at the 60-day stage (Figure S1). Using β-diversity, we determined that the mycelial bacterial community structure of inoculated AM fungi was different at different time stages (Figure 2c), and the α-diversity of AM fungi reached a stable state at 60 days (Figure 2a,b). Inoculation of AM fungi treatment simultaneously increased bacterial network complexity and stability (Table S4). During the whole cultivation process, the bacterial community showed a stable state at 60 days (Figure 2). In the 60–90-day phase, hyphosphere microbial communities became more similar (Figure 3). Thus, AM fungi accelerate the recovery of bacterial communities and establish stable communities in about 60 days.

AM fungi inoculation exhibited no statistically significant impact on the time taken for bacterial communities to stabilize in comparison with non-inoculated AM fungi treatment, but AM fungi treatment exhibited a higher diversity index at each time stage compared to non-inoculated AM fungi treatment (Figure 2a,b). Inoculation of AM fungi treatment simultaneously increased bacterial network complexity and stability (Table S2). Bacterial communities exhibiting higher phylogenetic diversity are characterized by greater stability, as indicated by their ability to resist ecological disturbance [30,38]. The inoculation with AM fungi has been demonstrated to induce alterations in the composition of carbon components, which in turn, exert a profound influence on the surrounding microbial communities. On the other hand, the existence and composition of particular bacterial communities in the hyphosphere can affect the AM fungi communities, thereby adding to the complexity of the assembly of below-ground communities [39]. Therefore, by promoting bacterial community diversity, AM fungi may enhance their resistance to environmental stressors, contributing to the long-term stability of ecosystems.

Different soil bacteria taxa and functional groups are likely subject to various community assembly mechanisms because of their specific ecological attributes and functional traits [40]. It has been established that AM fungi exudates contain organic acids and sugars [11], for which the supply of carbon and energy to hyphal-associated bacteria is essential for their growth and metabolism [34,41], and this effect results in a large quantity of bacteria being recruited by AM fungi hyphae [9,17]. Inoculation with AM fungi enhances deterministic processes for bacterial community assembly. Substances secreted by hyphae, such as fructose, glucose, and trehalose, attract specific microbial communities in a directional manner and gradually stabilize over time. This study revealed that AM fungi can accelerate the stabilization time of interhyphal microorganisms, indirectly demonstrating that this is a deterministic process. Compared with non-inoculated AM fungi, the proportion of deterministic processes increased from 30 days to 60 days after inoculation of AM fungi (Figure 4). Deterministic processes form microbial communities with appropriate functional redundancy and stable interaction networks, which can both resist invasion and recover from disturbances. Deterministic processes are characterized by the predominance of ecological sorting, whereby abiotic factors and biotic factors comprise distinct niches that exert a significant impact on community assembly [42]. Stochastic processes have demonstrated that species with considerably overlapping niches can co-occur, provided that their respective competitive abilities are similar and that random changes are not linked to environmental fitness [43]. The increased influence of deterministic processes on bacterial assembly may be attributed to elevated environmental selection pressures and heightened biological interactions [43,44]. It has been established that the intricate influence of AM fungi on soil bacterial communities is attributable to the nutrients they introduce, which in turn, engender complex alterations in community dynamics. Correlation analysis showed a significant influence of phosphorus (P) and carbon (C) availability on alterations in the bacterial community of inoculated AM fungi (Figure 5a,b).

The inoculated AM fungi treatment demonstrated a greater number of key species in comparison to the non-inoculated AM fungi treatment (Figure 3, Tables S5–S10). *Actinobacteriota*, *Gemmatimonadota*, *Patescibacteria*, *Chloroflexi*, *Bacteroidota*, *Firmicutes*, and *Patescibacteria* were the key nodes of the network of inoculated AM fungi. These species have been demonstrated to perform vital functions in the establishment and operation of microbial structures associated with plants [45,46,47]. They have the capacity to facilitate the transformation of organic nutrients into forms that are more readily absorbable by plants. Specifically, the role of these microbes is to activate soil nutrients, such as N and P, facilitating their recycling [48,49,50,51]. *Actinobacteriota* and *Gemmatimonadota* were significantly correlated with soil properties, both physical and chemical, and with bacterial stability indices under the treatment with AM fungi inoculation (Figure 5d), and *Actinobacteriota* and *Gemmatimonadota* were known as plant probiotics that benefit plant growth and communities. They play an integral role in the promotion of plant growth and the participation in the nitrogen cycle. *Actinobacteriota* also mineralize organic nitrogen, increasing soil ammonium. They can also solubilize insoluble phosphates, enhancing plant-available phosphorus. *Gemmatimonadota* aids in cycling carbon, nitrogen, and phosphorus in soil. They break down complex organic carbon into simpler compounds. They also transform nitrogen, including nitrification, denitrification, and ammonification. They also increase the availability of phosphorus by breaking down insoluble phosphates with organic acids. Furthermore, they have been shown to increase the capacity of plants to acclimatize to environmental changes and to fortify their defenses against pathogens [52]. The soil environment is more favorable for plant growth following inoculation with AM fungi (Table 1), and more beneficial bacteria are attracted to the plants. Conversely, non-inoculated AM fungi are more susceptible to colonization by alternative pathogenic bacteria [53].

## 5. Limitations of the Experimental Design

Although this experiment examined the dynamics of intermycelial bacterial community formation following inoculation with AM fungi, the use of one plant species (maize) and one arbuscular mycorrhizal fungi strain (*Rhizophagus irregularis*) resulted in a limited number of treatments. This limits the generalizability of the findings to broader scenarios. Future research could explore changes in hyphal-interacting microbial communities associated with a broader range of plant species and different types of AM fungi.

## 6. Conclusions

The non-destructive continuous sampling method used in this study allowed us to compare the hyphosphere bacterial community of AM fungi (*Rhizophagus irregularis*) at different time points. Concerning the AM fungal hyphosphere bacterial community, as expected, inoculation of corn plants with AM fungi caused significant changes in the bacterial community and its composition, both of which were stable after 60 days. During the 60–90-day phase, the hyphosphere microbial community became more similar. Inoculation with AM fungi simultaneously increased AM fungal network complexity and stability, thereby facilitating the formation of stable ecosystems. The inoculation of AM fungi has been demonstrated to promote deterministic processes within the assemblage of hyposphere bacterial communities. The *Actinobacteriota* and *Gemmatimonadota* were significantly associated with community stability and were significantly affected by available phosphorus and carbon. This study provides a more comprehensive understanding of the mechanisms underlying the composition of soil microbial community assemblages. Moreover, this study provides a scientific framework with which to elucidate the mechanisms of response exhibited by hyposphere bacterial communities in response to AM fungi.

## Figures and Tables

**Table 1:** The effects of AM fungi inoculation on the biomass at 90 days.

Treatment	Shoot Dry Weight (g·pot ^−1^)	Root Dry Weight (g·pot ^−1^)
–M	13.3 ± 1.8 b	3.2 ± 0.3 a
+M	20.5 ± 0.5 a	4.3 ± 0.6 a

Note: –M represents non-inoculated AM fungi treatment; +M represents inoculated AM fungi treatment. Different lowercase letters indicate significant differences (*p* < 0.05) at different soil treatments with Duncan’s multiple range test.

## Data Availability

The original contributions presented in this study are included in the article/Supplementary Material. Further inquiries can be directed to the corresponding authors.

## Supplementary Materials

The following supporting information can be downloaded at: https://www.mdpi.com/article/10.3390/jof11100739/s1, Table S1: The mycorrhizal infection of the different treatments. Table S2: Topological properties of molecular ecological networks in different treatments. Table S3: Indicators of soil carbon (C), nitrogen (N), and organic carbon (TOC). Table S4: Soil physical and chemical indicators. Table S5: Key bacterial communities at 30 days of maize growth under non-inoculated AM fungi treatment. Table S6: Key bacterial communities at 60 days of maize growth under non-inoculated AM fungi treatment. Table S7: Key bacterial communities at 90 days of maize growth under non-inoculated AM fungi treatment. Table S8: Key bacterial communities at 30 days of maize growth under inoculated AM fungi treatment. Table S9: Key bacterial communities at 60 days of maize growth under inoculated AM fungi treatment. Table S10: Key bacterial communities at 90 days of maize growth under inoculated AM fungi treatment. Figure S1: The contents of total P, Fe, Ca and Mg in the soil as affected by mycorrhizal inoculation. Figure S2: Interaction frequency of main taxa of maize bacterial community with the chord diagrams. Figure S3: Alpha diversity analysis of bacterial communities. Figure S4: The co-occurrence networks of bacterial communities. a, b and c represent non -inoculated treatment; d, e and f represent inoculated with AM fungi treatment. Figure S5: Correlation analysis of the soil parameters related with AM fungi hyphosphere bacterial communities. (a) Non-inoculated treatment; (b) Inoculated with AM fungi treatment. Figure S6: Correlation analysis of the soil related with bacterial communities. (a) Non-inoculated treatment; (b) Inoculated with AM fungi treatment.
