# Supplementary material for: Effect of Inoculation with Arbuscular Mycorrhizal Fungi (Rhizophagus irregularis BGC AH01) on the Soil Bacterial Community Assembly
Source: J Fungi (Basel). 2025 Oct 15;11(10):739. doi: 10.3390/jof11100739 (PMC12565222; doi:10.3390/jof11100739)
Supplement: Supplementary file 1 [file jof-11-00739-s001.zip › jof-3822384-supplementary.pdf]

Effect of Inoculation with Arbuscular Mycorrhizal Fungi (*Rhizophagus irregularis* BGC AH01) on the Soil Bacterial Community Assembly

Xueli Wang [1], Xuemin Jing [2,3], Yan Wang [4], Youran Ma [5], Xiangyang Shu [1], Wei Fu [2], Shuping Xing [6], Weijia Liu [7], Qinxin Ye [7], Yalan Zhu [1], Ping Ren [1], Xin Zhang [2,8,*], Baodong Chen [2,8] and Xia Wang [1,2,*]

[1] The Key Laboratory of Land Resources Evaluation and Monitoring in Southwest China, College of Geography and Resources, Sichuan Normal University, Chengdu 610066, China; wangxueli@stu.sicnu.edu.cn (X.W.); xyshu@sicnu.edu.cn (X.S.); zhuyalan2025@163.com (Y.Z.); renping@sicnu.edu.cn (P.R.)

[2] State Key Laboratory for Ecological Security of Regions and Cities, Research Center for Eco-Environmental Sciences, Chinese Academy of Sciences, Beijing 100085, China; jxm6452@163.com (X.J.); weifu@rcees.ac.cn (W.F.); bdchen@rcees.ac.cn (B.C.)

[3] Heilongjiang Provincial Key Laboratory of Ecological Restoration and Resource Utilization for Cold Region, School of Life Sciences, Heilongjiang University, Harbin 150080, China

[4] School of Chemistry and Materials Science, Sichuan Normal University, Chengdu 610066, China; wangyanwangyan@stu.sicnu.edu.cn

[5] China Aerospace Construction Group Co., Ltd., Beijing 100071, China; superyoyo9938@163.com

[6] Information Center of Ministry of Ecology and Environment, Beijing100029, China; stellar_xsp@sina.com

[7] Institute of Agricultural Bioenvironment and Energy, Chengdu Academy of Agriculture and Forestry Sciences, Chengdu 611130, China; liuweijia27@163.com (W.L.); yeqinxin123@163.com (Q.Y.)

[8] University of Chinese Academy of Sciences, Beijing 100049, China

* Correspondence: xinzhang@rcees.ac.cn (X.Z.); wangxia114@mails.ucas.ac.cn (X.W.)

Supplementary Material List

Table

Table S1 The mycorrhizal infection of the different treatments.

Table S2 Topological properties of molecular ecological networks in different treatments.

Table S3 Indicators of soil carbon (C), nitrogen (N), and organic carbon (TOC).

Table S4 Soil physical and chemical indicators.

Table S5 Key bacterial communities at 30 days of maize growth under non-inoculated AM fungi treatment.

Table S6 Key bacterial communities at 60 days of maize growth under non-inoculated AM fungi treatment.

Table S7 Key bacterial communities at 90 days of maize growth under non-inoculated AM fungi treatment.

Table S8 Key bacterial communities at 30 days of maize growth under inoculated AM fungi treatment.

Table S9 Key bacterial communities at 60 days of maize growth under inoculated AM fungi treatment.

Table S10 Key bacterial communities at 90 days of maize growth under inoculated AM fungi treatment.

Figure

Figure S1 The contents of total P, Fe, Ca and Mg in the soil as affected by mycorrhizal inoculation.

Figure S2 Interaction frequency of main taxa of maize bacterial community with the chord diagrams.

Figure S3 Alpha diversity analysis of bacterial communities.

Figure S4 The co-occurrence networks of bacterial communities. a, b and c represent non -inoculated treatment; d, e and f represent inoculated with AM fungi treatment.

Figure S5 Correlation analysis of the soil parameters related with AM fungi hyphosphere bacterial communities. (a) Non-inoculated treatment; (b) Inoculated with AM fungi treatment.

Figure S6 Correlation analysis of the soil related with bacterial communities. (a) Non-inoculated treatment; (b) Inoculated with AM fungi treatment.

Table S1 The mycorrhizal infection of the different treatments.

Treatment	M%	m%	a%	A%
– M	3	3	1.28	1.28
+ M	85.1	85.1	55.2	55.2

Notes: –M represent non-inoculated treatment; +M represent inoculated AM fungi treatment; M %: the mycorrhizal infection intensity of the whole root system; m %: Characterize the frequency and intensity of fungal infection in all infected root segments; a %: arbuscular abundance of infected root segments; A %: Arbuscular abundance of the whole root system.

Table S2 Topological properties of molecular ecological networks in different treatments.

Network type	Node number	Edge number	Average degree	Modula-rity	Number of positive correlations	Number of negative correlations
A – M	111	101	1.8	0.95	98.02%	1.98%
B – M	242	210	1.8	0.98	92.38%	0.41%
C – M	300	372	2.5	0.97	96.49%	3.51%
A + M	256	319	2.5	0.98	99.22%	0.78%
B + M	389	441	2.3	0.98	89.8%	10.2%
C + M	389	483	2.5	0.96	95.16%	4.84%

Notes: A–M represent 30 days non-inoculated AM fungi treatment. B–M represent 60 days non-inoculated AM fungi treatment. C–M represent 90 days non-inoculated AM fungi treatment. A+M represent 30 days inoculated AM fungi treatment. B+M represent 60 days inoculated AM fungi treatment. C+M represent 90 days inoculated AM fungi treatment.

Table S3 Indicators of soil carbon (C), nitrogen (N), and organic carbon (TOC).

Treatment	C (mg g^{-1})	N (mg g^{-1})	TOC (mg g^{-1})
A – M	0.1 ± 0.004 a	0.0036 ± 0.0001 b	0.0687 ± 0.001 b
B – M	0.1 ± 0.003 a	0.0038 ± 0.0001 ab	0.0645 ± 0.001 ab
C – M	0.1016 ± 0.002 a	0.0035 ± 0.0001 ab	0.0688 ± 0.002 ab
A + M	0.1019 ± 0.002 a	0.0039 ± 0.0002 ab	0.0701 ± 0.001 b
B + M	0.1031 ± 0.002 a	0.0037 ± 0.0001 ab	0.0651 ± 0.001 ab
C + M	0.1018 ± 0.003 a	0.0034 ± 0.0001 a	0.0664 ± 0.002 a

Notes:A–M represent 30 days non-inoculated AM fungi treatment. B–M represent 60 days non-inoculated AM fungi treatment. C–M represent 90 days non-inoculated AM fungi treatment. A+M represent 30 days inoculated AM fungi treatment. B+M represent 60 days inoculated AM fungi treatment. C+M represent 90 days inoculated AM fungi treatment.

Table S4 Soil physical and chemical indicators.

Treatment	pH	AP (mg kg^{-1})	CEC (cmol $^{+}$ kg^{-1})
− M	7.9233 ± 0.04 a	23.4 ± 0.7 a	8.6 ± 0.57 a
+ M	7.9283 ± 0.05 a	15.7 ± 1.4 b	7.6 ± 0.63 b

Notes: –M represent non-inoculated treatment; +M represent inoculated AM fungi treatment.

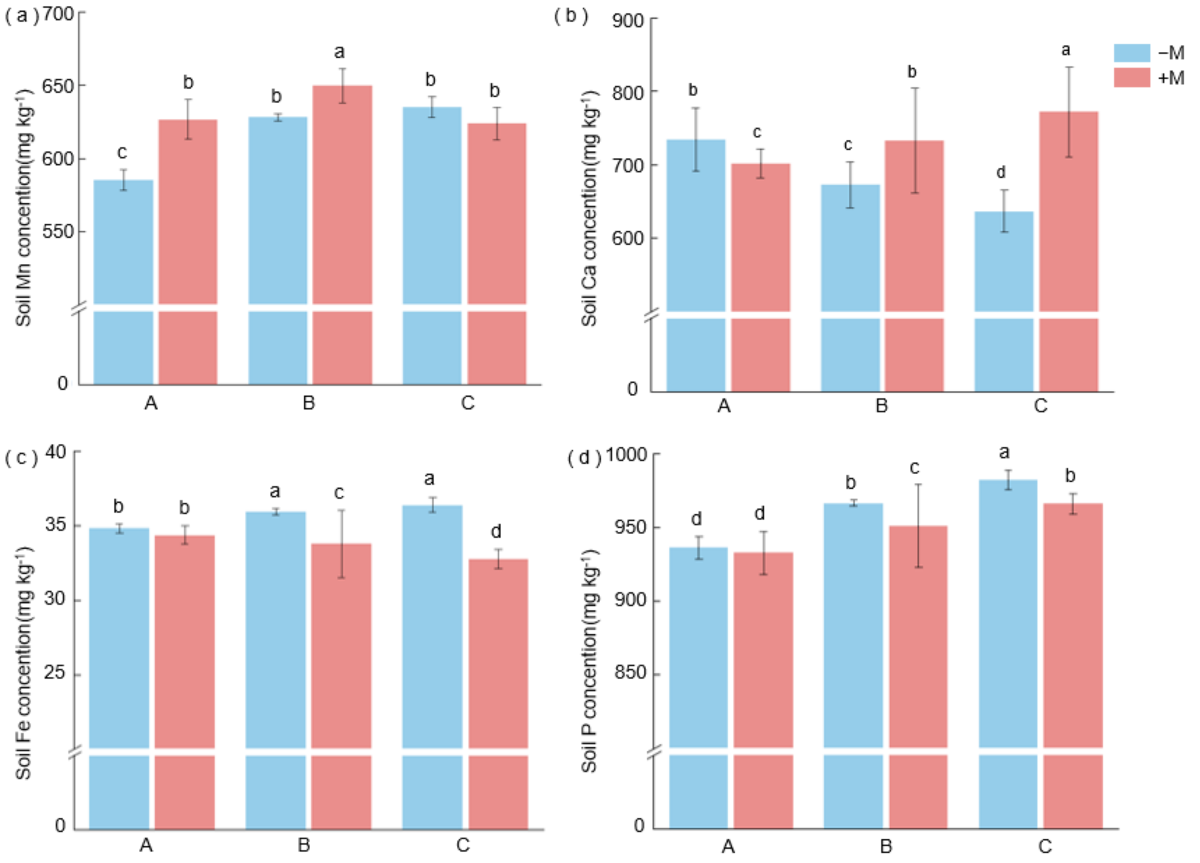

Figure S1. The contents of total P, Fe, Ca and Mg in the soil as affected by mycorrhizal inoculation. (–M represent non-inoculated treatment; A–M represent 30 days non-inoculated AM fungi treatment. B–M represent 60 days non-inoculated AM fungi treatment. C–M represent 90 days non-inoculated AM fungi treatment. A+M represent 30 days inoculated AM fungi treatment. B+M represent 60 days inoculated AM fungi treatment. C+M represent 90 days inoculated AM fungi treatment.   Different lowercase letters indicate significant differences ($p < 0.05$) at different soil treatments with the Duncan's multiple range test.）

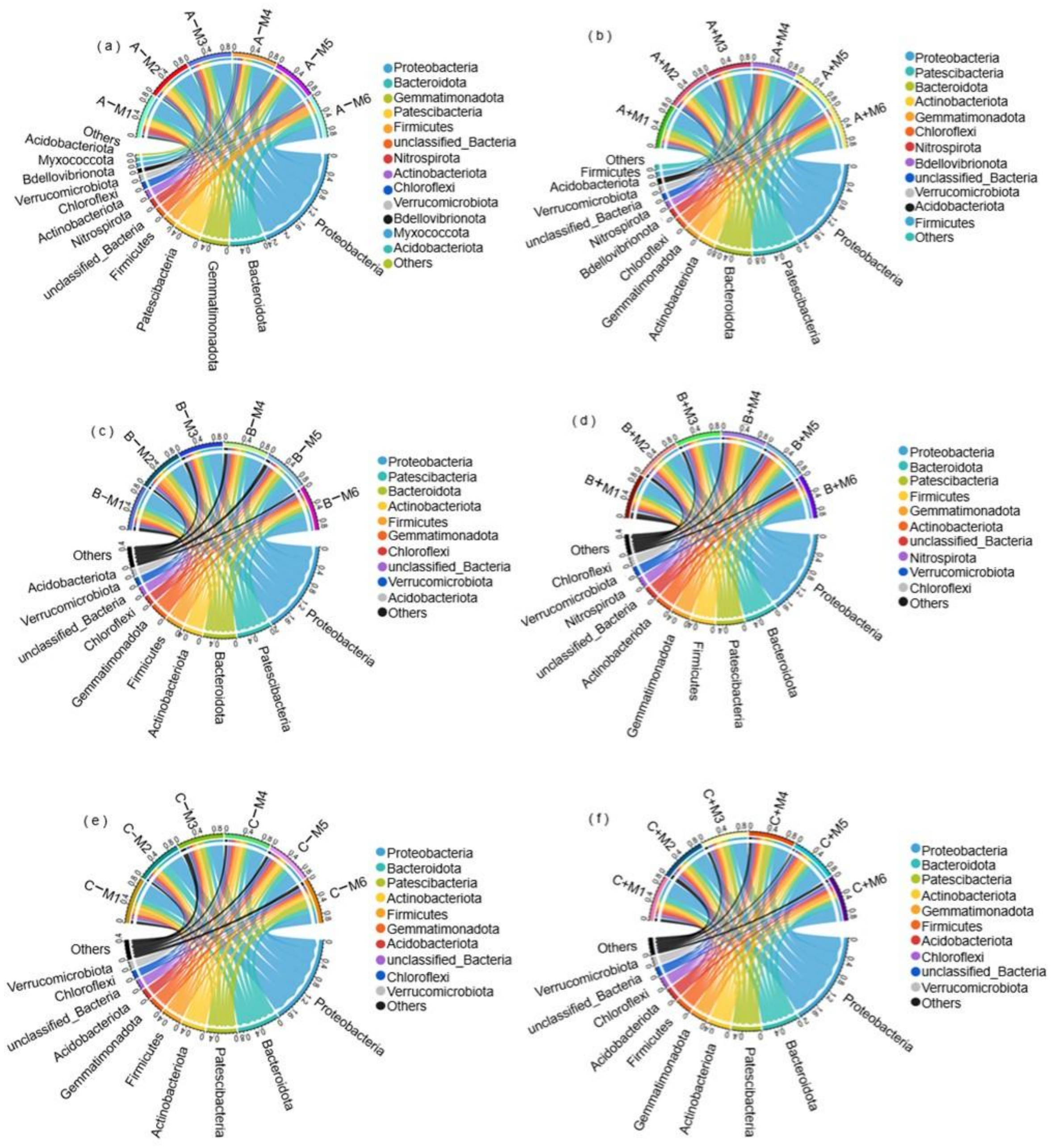

Figure S2. Interaction frequency of main taxa of maize bacterial community with the chord diagrams. A–M represent 30 days non-inoculated AM fungi treatment. B–M represent 60 days non-inoculated AM fungi treatment. C–M represent 90 days non-inoculated AM fungi treatment. A+M represent 30 days inoculated AM fungi treatment. B+M represent 60 days inoculated AM fungi treatment. C+M represent 90 days inoculated AM fungi treatment.)

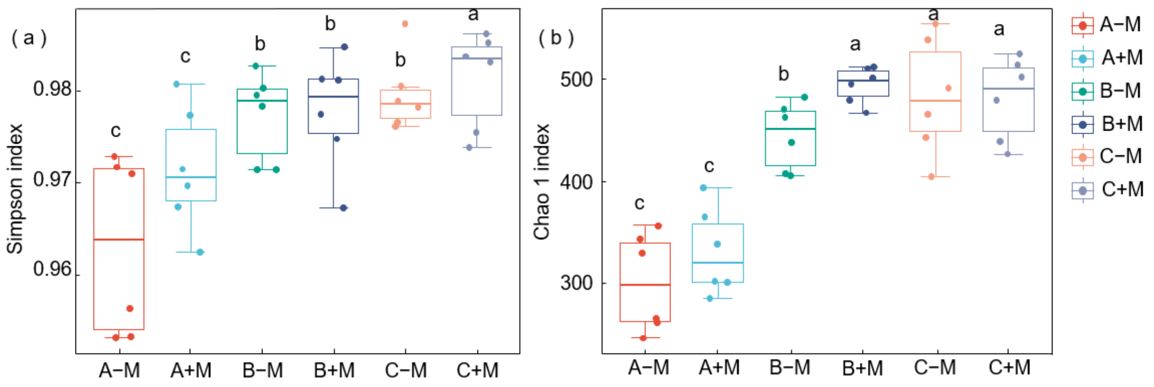

Figure S3. Alpha diversity analysis of bacterial communities. (A–M represent 30 days non-inoculated AM fungi treatment. B–M represent 60 days non-inoculated AM fungi treatment. C–M represent 90 days non-inoculated AM fungi treatment. A+M represent 30 days inoculated AM fungi treatment. B+M represent 60 days inoculated AM fungi treatment. C+M represent 90 days inoculated AM fungi treatment.)

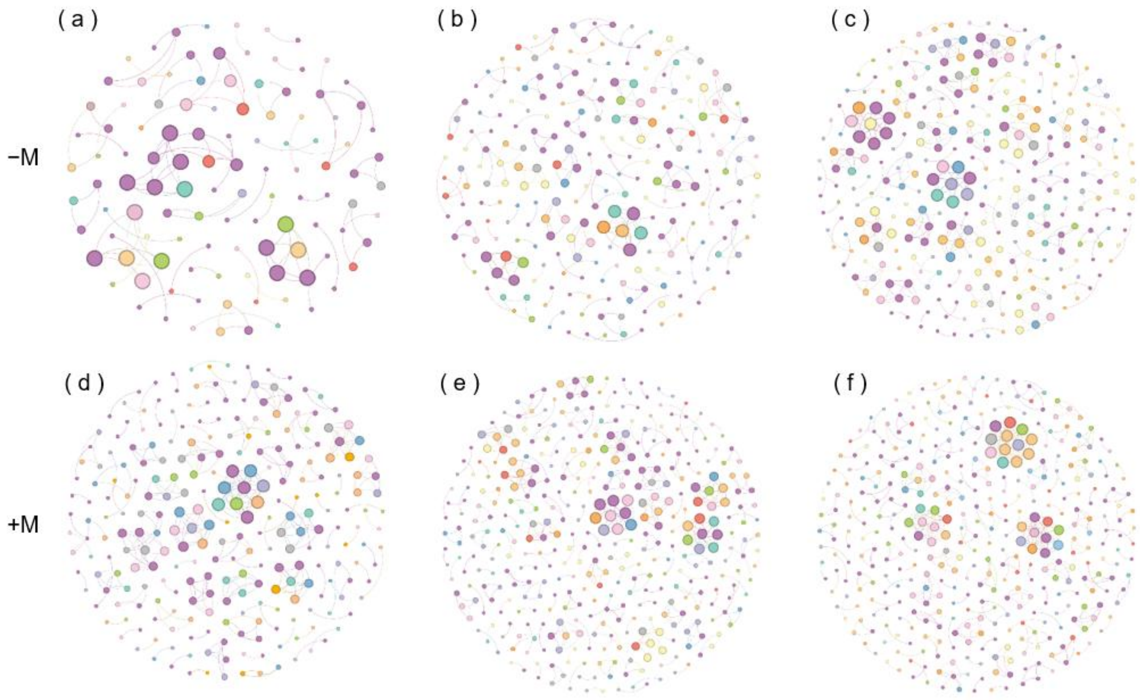

Figure S4. The co-occurrence networks of bacterial communities. a, b and c represent non -inoculated treatment; d, e and f represent inoculated with AM fungi treatment. (A means the time of plant growth 30 days; B means the time of plant growth 60 days; C means the time of plant growth 90 days.)

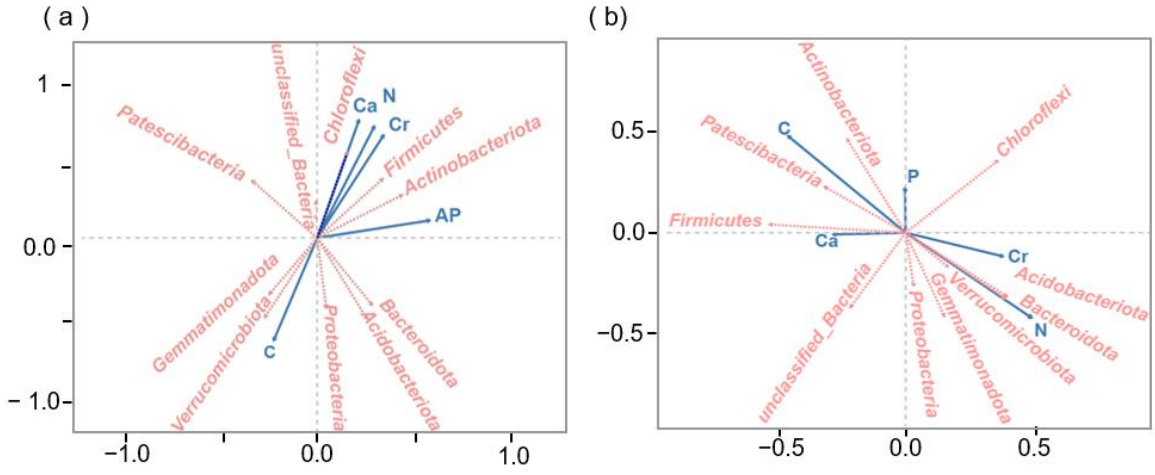

Figure S5. Correlation analysis of the soil parameters related with AM fungi hyphosphere bacterial communities. (a) Non-inoculated treatment; (b) Inoculated with AM fungi treatment.

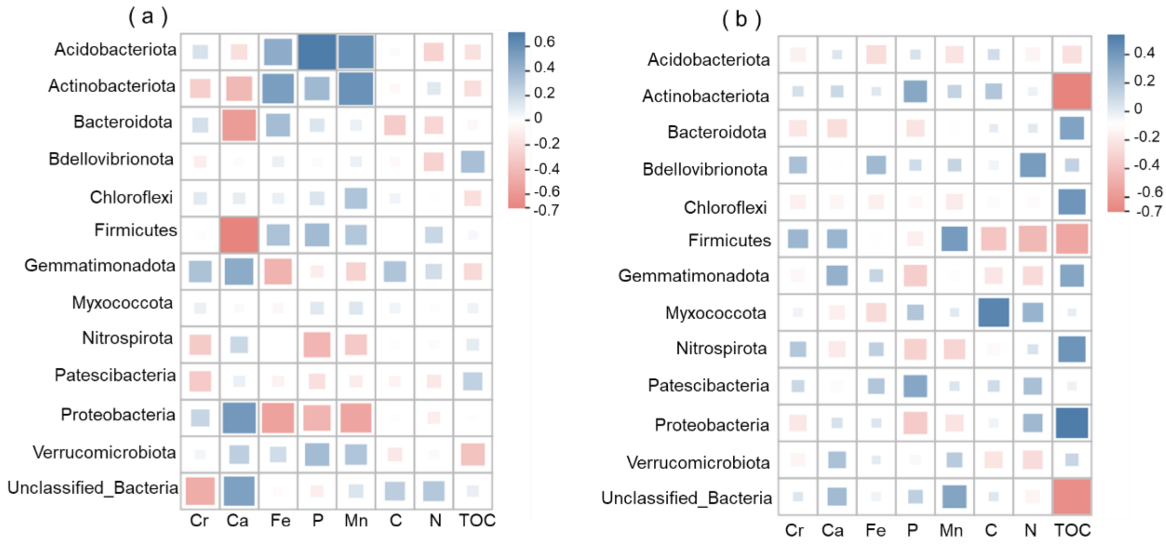

Figure S6. Correlation analysis of the soil related with bacterial communities. (a) Non-inoculated treatment; (b) Inoculated with AM fungi treatment.

Table S5 Key bacterial communities at 30 days of maize growth under non-inoculated AM fungi treatment.

type	Kingdom	Phylum	Class	Order	Family	Genus	Species
Connectors	Bacteria	Nitrospirota	Nitrospiria	Nitrospirales	Nitrospiraceae	Nitrospira	unclassified_Nitrospira
Connectors	Bacteria	Proteobacteria	Alphaproteobacteria	Rhizobiales	Beijerinckiaceae	unclassified_Beijerinckiaceae	unclassified_Beijerinckiaceae
Connectors	Bacteria	Myxococcota	Myxococcia	Myxococcales	Myxococcaceae	unclassified_Myxococcaceae	unclassified_Myxococcaceae
Connectors	Bacteria	Bacteroidota	Rhodothermia	Rhodothermales	Rhodothermaceae	unclassified_Rhodothermaceae	unclassified_Rhodothermaceae
Connectors	Bacteria	Myxococcota	Myxococcia	Myxococcales	Anaeromyxobacteraceae	Anaeromyxobacter	unclassified_Anaeromyxobacter
Connectors	Bacteria	Proteobacteria	Alphaproteobacteria	Sphingomonadales	Sphingomonadaceae	Sphingomonas	unclassified_Sphingomonas
Connectors	Bacteria	Gemmatimonadota	Gemmatimonadetes	Gemmatimonadales	Gemmatimonadaceae	Gemmatimonas	unclassified_Gemmatimonas
Connectors	Bacteria	Proteobacteria	Alphaproteobacteria	Caulobacterales	Caulobacteraceae	Phenylobacterium	unclassified_Phenylobacterium
Connectors	Bacteria	unclassified_Bacteria	unclassified_Bacteria	unclassified_Bacteria	unclassified_Bacteria	unclassified_Bacteria	unclassified_Bacteria
Connectors	Bacteria	Firmicutes	Bacilli	Bacillales	Bacillaceae	Bacillus	unclassified_Bacillus
Connectors	Bacteria	Bacteroidota	Bacteroidia	Cytophagales	Microscillaceae	unclassified_Microscillaceae	unclassified_Microscillaceae
Connectors	Bacteria	Nitrospirota	Nitrospiria	Nitrospirales	Nitrospiraceae	Nitrospira	unclassified_Nitrospira
Connectors	Bacteria	Actinobacteriota	Actinobacteria	Micrococcales	Microbacteriaceae	unclassified_Microbacteriaceae	unclassified_Microbacteriaceae
Connectors	Bacteria	Proteobacteria	Gammaproteobacteria	Steroidobacterales	Steroidobacteraceae	Steroidobacter	Steroidobacter_agariperforans
Connectors	Bacteria	Actinobacteriota	Acidimicrobiia	Microtrichales	Iamiaceae	Iamia	unclassified_Iamia

Connectors	Bacteria	Gemmatimonadota	Longimicrobia	Longimicrobiales	Longimicrobiaceae	Longimicrobium	unclassified_Longimicrobium
Connectors	Bacteria	Gemmatimonadota	Longimicrobia	Longimicrobiales	Longimicrobiaceae	unclassified_Longimicrobiaceae	unclassified_Longimicrobiaceae
Connectors	Bacteria	Verrucomicrobiota	Verrucomicrobiae	Pedosphaerales	Pedosphaeraceae	unclassified_Pedosphaeraceae	unclassified_Pedosphaeraceae
Connectors	Bacteria	Bacteroidota	Bacteroidia	Chitinophagales	Chitinophagaceae	Flavisolibacter_sp.	unclassified_Flavisolibacter_sp.
Connectors	Bacteria	Chloroflexi	Chloroflexia	Chloroflexales	Herpetosiphonaceae	Herpetosiphon	uncultured_Chloroflexi_bacterium
Connectors	Bacteria	Gemmatimonadota	Longimicrobia	Longimicrobiales	Longimicrobiaceae	Longimicrobium	unclassified_Longimicrobium
Connectors	Bacteria	Bdellovibrionota	Bdellovibrionia	Bdellovibrionales	Bdellovibrionaceae	Bdellovibrio	unclassified_Bdellovibrio
Connectors	Bacteria	Gemmatimonadota	Longimicrobia	Longimicrobiales	Longimicrobiaceae	YC_ZSS_LKJ147	unclassified_YC_ZSS_LKJ147
Connectors	Bacteria	Proteobacteria	Gammaproteobacteria	Steroidobacterales	Steroidobacteraceae	Steroidobacter	unclassified_Steroidobacter
Connectors	Bacteria	Chloroflexi	Chloroflexia	Thermomicrobiales	AKYG1722	unclassified_AKYG1722	unclassified_AKYG1722
Connectors	Bacteria	Proteobacteria	Gammaproteobacteria	Pseudomonadales	Moraxellaceae	Cavicella	unclassified_Cavicella
Connectors	Bacteria	Bacteroidota	Bacteroidia	Sphingobacteriales	Sphingobacteriaceae	unclassified_Sphingobacteriaceae	unclassified_Sphingobacteriaceae
Connectors	Bacteria	Actinobacteriota	Acidimicrobiia	unclassified_Acidimicrobiia	uncultured_Acidimicrobiia_bacterium	unclassified_Acidimicrobiia	unclassified_Acidimicrobiia
Connectors	Bacteria	Actinobacteriota	Actinobacteria	Micrococcales	Cellulomonadaceae	Cellulomonas	unclassified_Cellulomonas
Connectors	Bacteria	Bacteroidota	Bacteroidia	Cytophagales	Microscillaceae	unclassified_Microscillaceae	uncultured_Flexibacteraceae

							_bacterium
Connectors	Bacteria	Firmicutes	Clostridia	Lachnospirales	Lachnospiraceae	Lachnospiraceae_NK4A136_group	unclassified_Lachnospiraceae_NK4A136_group
Connectors	Bacteria	Proteobacteria	Alphaproteobacteria	Sphingomonadales	Sphingomonadaceae	unclassified_Sphingomonadaceae	unclassified_Sphingomonadaceae
Connectors	Bacteria	Proteobacteria	Alphaproteobacteria	Caedibacterales	Caedibacteraceae	Candidatus_Nucleicultrix	unclassified_Candidatus_Nucleicultrix
Connectors	Bacteria	Proteobacteria	Alphaproteobacteria	Rhizobiales	Rhizobiaceae	Allorhizobium_Neorhizobium_Pararhizobium_Rhizobium	unclassified_Allorhizobium_Neorhizobium_Pararhizobium_Rhizobium
Module hubs	Bacteria	Bdellovibrionota	Bdellovibrionia	Bdellovibrionales	Bdellovibrionaceae	Bdellovibrio	unclassified_Bdellovibrio
Module hubs	Bacteria	Bacteroidota	Bacteroidia	Sphingobacteriales	KD3_93	unclassified_KD3_93	unclassified_KD3_93
Module hubs	Bacteria	Firmicutes	Bacilli	Bacillales	Bacillaceae	Bacillus	unclassified_Bacillus
Module hubs	Bacteria	Proteobacteria	Alphaproteobacteria	Sphingomonadales	Sphingomonadaceae	Sphingomonas	unclassified_Sphingomonas
Module hubs	Bacteria	Proteobacteria	Gammaproteobacteria	Burkholderiales	Burkholderiaceae	Lautropia	unclassified_Lautropia
Module hubs	Bacteria	Gemmatimonadota	Gemmatimonadetes	Gemmatimonadales	Gemmatimonadaceae	unclassified_Gemmatimonadaceae	unclassified_Gemmatimonadaceae
Module hubs	Bacteria	Chloroflexi	Chloroflexia	Thermomicrobiales	Thermomicrobiaceae	Nitrolancea	unclassified_Nitrolancea
Module hubs	Bacteria	Bacteroidota	Bacteroidia	Sphingobacteriales	Sphingobacteriaceae	Pedobacter	unclassified_Pedobacter

Table S6 Key bacterial communities at 60 days of maize growth under non-inoculated AM fungi treatment.

type	Kingdom	Phylum	Class	Order	Family	Genus	Species
Connectors	Bacteria	Proteobacteria	Alphaproteobacteria	Rhizobiales	Rhizobiaceae	unclassified_Rhizobiaceae	unclassified_Rhizobiaceae
Connectors	Bacteria	Chloroflexi	Chloroflexia	Thermomicrobiales	AKYG1722	uncultured_Alphaproteobacteria_bacterium	uncultured_Alphaproteobacteria_bacterium
Connectors	Bacteria	Bacteroidota	Bacteroidia	Bacteroidales	Muribaculaceae	unclassified_Muribaculaceae	unclassified_Muribaculaceae
Connectors	Bacteria	Actinobacteriota	Actinobacteria	Propionibacteriales	Nocardioidaceae	Nocardioides	unclassified_Nocardioides
Connectors	Bacteria	Firmicutes	Clostridia	Lachnospirales	Lachnospiraceae	Lachnospiraceae_NK4A136_group	unclassified_Lachnospiraceae_NK4A136_group
Connectors	Bacteria	Bacteroidota	Bacteroidia	Bacteroidales	Muribaculaceae	unclassified_Muribaculaceae	unclassified_Muribaculaceae
Connectors	Bacteria	Firmicutes	Clostridia	Lachnospirales	Lachnospiraceae	unclassified_Lachnospiraceae	unclassified_Lachnospiraceae
Connectors	Bacteria	Firmicutes	Clostridia	Lachnospirales	Lachnospiraceae	unclassified_Lachnospiraceae	unclassified_Lachnospiraceae
Connectors	Bacteria	Actinobacteriota	Acidimicrobiia	unclassified_Acidimicrobiia	uncultured_Acidimicrobiia_bacterium	unclassified_Acidimicrobiia	unclassified_Acidimicrobiia
Connectors	Bacteria	Proteobacteria	Gammaproteobacteria	Steroidobacterales	Steroidobacteraceae	Steroidobacter	Steroidobacter_agariperforans
Connectors	Bacteria	Proteobacteria	Alphaproteobacteria	Rhizobiales	Hyphomicrobiaceae	unclassified_Hyphomicrobiaceae	unclassified_Hyphomicrobiaceae
Connectors	Bacteria	Bacteroidota	Bacteroidia	Bacteroidales	Prevotellaceae	Alloprevotella	uncultured_Bacteroidales_bacterium
Connectors	Bacteria	Bacteroidota	Bacteroidia	Cytophagales	Microscillaceae	unclassified_Microscillaceae	uncultured_Flexibacteraceae_bacterium

Connectors	Bacteria	Proteobacteria	Alphaproteobacteria	Sphingomonadales	Sphingomonadaceae	Altererythrobacter	unclassified_Altererythrobacter
Connectors	Bacteria	Verrucomicrobiota	Verrucomicrobiae	Pedosphaerales	Pedosphaeraceae	unclassified_Pedosphaeraceae	unclassified_Pedosphaeraceae
Connectors	Bacteria	Bacteroidota	Bacteroidia	Bacteroidales	Muribaculaceae	unclassified_Muribaculaceae	unclassified_Muribaculaceae
Connectors	Bacteria	Bacteroidota	Bacteroidia	Bacteroidales	Muribaculaceae	unclassified_Muribaculaceae	unclassified_Muribaculaceae
Connectors	Unassigned	Unassigned	Unassigned	Unassigned	Unassigned	Unassigned	Unassigned
Modulehubs	Bacteria	Proteobacteria	Alphaproteobacteria	Rhizobiales	Beijerinckiaceae	Microvirga	unclassified_Microvirga
Modulehubs	Bacteria	Gemmatimonadota	Gemmatimonadetes	Gemmatimonadales	Gemmatimonadaceae	Gemmatimonas	unclassified_Gemmatimonas
Modulehubs	Bacteria	Proteobacteria	Gammaproteobacteria	Burkholderiales	Nitrosomonadaceae	Nitrosomonas	unclassified_Nitrosomonas
Modulehubs	Bacteria	Myxococcota	Myxococcia	Myxococcales	Anaeromyxobacteraceae	Anaeromyxobacter	unclassified_Anaeromyxobacter
Modulehubs	Bacteria	Proteobacteria	Alphaproteobacteria	Rhizobiales	Rhizobiaceae	Ensifer	unclassified_Ensifer
Modulehubs	Bacteria	Proteobacteria	Alphaproteobacteria	Rhizobiales	Rhizobiales_Incertae_Sedis	unclassified_Rhizobiales_Incertae_Sedis	unclassified_Rhizobiales_Incertae_Sedis
Modulehubs	Bacteria	Gemmatimonadota	Gemmatimonadetes	Gemmatimonadales	Gemmatimonadaceae	Gemmatimonas	unclassified_Gemmatimonas
Modulehubs	Bacteria	Firmicutes	Clostridia	Clostridia_UCG_014	unclassified_Clostridia_UCG_014	unclassified_Clostridia_UCG_014	unclassified_Clostridia_UCG_014
Modulehubs	Bacteria	Firmicutes	Clostridia	Oscillospirales	Oscillospiraceae	unclassified_Oscillospiraceae	unclassified_Oscillospiraceae
Modulehub	Bacteria	Patescibacter	Gracilibacteri	JGI_0000069_P22	unclassified_J	unclassified_JGI_0000069	unclassified_JGI_0000069_

s		ia	a		GI_0000069_P22	_P22	P22
Modulehubs	Bacteria	Gemmatimonadota	Gemmatimonadetes	Gemmatimonadales	Gemmatimonadaceae	Gemmatimonas	unclassified_Gemmatimonas
Modulehubs	Bacteria	Actinobacteriota	Acidimicrobiia	unclassified_Acidimicrobiia	uncultured_soil_bacterium	unclassified_Acidimicrobiia	unclassified_Acidimicrobiia
Modulehubs	Bacteria	Planctomycetota	Planctomycetes	Pirellulales	Pirellulaceae	Pirellula	unclassified_Pirellula
Modulehubs	Bacteria	Firmicutes	Clostridia	Lachnospirales	Lachnospiraceae	Lachnospiraceae_NK4A136_group	Lachnospiraceae_bacterium_COE1
Modulehubs	Bacteria	Bacteroidota	Bacteroidia	Bacteroidales	Prevotellaceae	Prevotellaceae_UCG_003	unclassified_Prevotellaceae_UCG_003
Modulehubs	Bacteria	Proteobacteria	Alphaproteobacteria	Sphingomonadales	Sphingomonadaceae	Sphingoaurantiacus	unclassified_Sphingoaurantiacus
Modulehubs	Bacteria	Firmicutes	Clostridia	Oscillospirales	Oscillospiraceae	Oscillibacter	unclassified_Oscillibacter
Modulehubs	Bacteria	Firmicutes	Clostridia	Lachnospirales	Lachnospiraceae	Lachnospiraceae_NK4A136_group	unclassified_Lachnospiraceae_NK4A136_group
Modulehubs	Bacteria	Proteobacteria	Alphaproteobacteria	Rhizobiales	Devosiaceae	unclassified_Devosiaceae	unclassified_Devosiaceae
Modulehubs	Bacteria	Firmicutes	Limnochordia	Limnochordales	Limnochordaceae	uncultured_compost_bacterium	uncultured_compost_bacterium
Modulehubs	Bacteria	Firmicutes	Limnochordia	Limnochordales	Limnochordaceae	uncultured_compost_bacterium	uncultured_compost_bacterium
Modulehubs	Bacteria	Bdellovibrionota	Bdellovibrionia	Bdellovibrionales	Bdellovibrionaceae	Bdellovibrio	unclassified_Bdellovibrio
Modulehubs	Bacteria	Actinobacteriota	Coriobacteriia	Coriobacteriales	Atopobiaceae	Olsenella	unclassified_Olsenella
Modulehubs	Bacteria	Proteobacteria	Alphaproteobacteria	Rhizobiales	Xanthobacteraceae	unclassified_Xanthobacteraceae	unclassified_Xanthobacteraceae

Modulehubs	Bacteria	unclassified_Bacteria	unclassified_Bacteria	unclassified_Bacteria	unclassified_Bacteria	unclassified_Bacteria	unclassified_Bacteria
Modulehubs	Bacteria	Proteobacteria	Alphaproteobacteria	Zavarziniales	unclassified_Zavarziniales	unclassified_Zavarziniales	unclassified_Zavarziniales
Modulehubs	Bacteria	Proteobacteria	Alphaproteobacteria	Rhodospirillales	Rhodospirillaceae	unclassified_Rhodospirillaceae	unclassified_Rhodospirillaceae
Modulehubs	Bacteria	Bacteroidota	Bacteroidia	Sphingobacteriales	KD3_93	unclassified_KD3_93	unclassified_KD3_93
Modulehubs	Bacteria	Actinobacteriota	Actinobacteria	Propionibacteriales	Nocardioidaceae	Nocardioides	unclassified_Nocardioides
Modulehubs	Bacteria	Proteobacteria	Alphaproteobacteria	Rhizobiales	Xanthobacteraceae	Pseudolabrys	unclassified_Pseudolabrys

Table S7 Key bacterial communities at 90 days of maize growth under non-inoculated AM fungi treatment.

type	Kingdom	Phylum	Class	Order	Family	Genus	Species
Connectors	Bacteria	Proteobacteria	Alphaproteobacteria	Sphingomonadales	Sphingomonadaceae	Sphingoaurantiacus	unclassified_Sphingoaurantiacus
Connectors	Bacteria	Chloroflexi	Chloroflexia	Thermomicrobiales	AKYG1722	unclassified_AKYG1722	unclassified_AKYG1722
Connectors	Bacteria	Actinobacteriota	Acidimicrobiia	unclassified_Acidimicrobiia	uncultured_Acidimicrobiia_bacterium	unclassified_Acidimicrobiia	unclassified_Acidimicrobiia
Connectors	Bacteria	Gemmatimonadota	Gemmatimonadetes	Gemmatimonadales	Gemmatimonadaceae	unclassified_Gemmatimonadaceae	unclassified_Gemmatimonadaceae
Connectors	Bacteria	Firmicutes	Clostridia	Oscillospirales	Oscillospiraceae	Colidextribacter	unclassified_Colidextribacter
Connectors	Bacteria	Bacteroidota	Bacteroidia	Bacteroidales	Muribaculaceae	unclassified_Muribaculaceae	unclassified_Muribaculaceae
Connectors	Bacteria	Firmicutes	Bacilli	RF39	unclassified_RF39	unclassified_RF39	unclassified_RF39
Connectors	Bacteria	Bacteroidota	Bacteroidia	Bacteroidales	Muribaculaceae	unclassified_Muribaculaceae	unclassified_Muribaculaceae
Connectors	Bacteria	Chloroflexi	Dehalococcoidia	SAR202_clade	uncultured_Chloroflexi_bacterium	uncultured_Chloroflexi_bacterium	uncultured_Chloroflexi_bacterium
Connectors	Bacteria	Nitrospirota	Nitrospiria	Nitrospirales	Nitrospiraceae	Nitrospira	unclassified_Nitrospira
Connectors	Bacteria	Acidobacteriota	Blastocatellia	Blastocatellales	Blastocatellaceae	Stenotrophobacter	uncultured_Acidobacteriaceae_bacterium
Connectors	Bacteria	Proteobacteria	Alphaproteobacteria	Sphingomonadales	Sphingomonadaceae	Sphingomonas	unclassified_Sphingomonas
Connectors	Bacteria	Firmicutes	Bacilli	Bacillales	Bacillaceae	Bacillus	unclassified_Bacillus
Connectors	Bacteria	Gemmatimonadota	Gemmatimonadetes	Gemmatimonadales	Gemmatimonadaceae	unclassified_Gemmatimonadaceae	unclassified_Gemmatimonadaceae
Connectors	Bacteria	Actinobacterio	Thermoleoph	Gaiellales	unclassified_Gaiel	unclassified_Gaiellales	unclassified_Gaiellales

		ta	ilia		lales		
Modulehubs	Bacteria	Acidobacteriota	Holophagae	Subgroup_7	uncultured_soil_bacterium	uncultured_soil_bacterium	uncultured_soil_bacterium
Modulehubs	Bacteria	Firmicutes	Bacilli	Paenibacillales	Paenibacillaceae	Paenibacillus	unclassified_Paenibacillus
Modulehubs	Bacteria	Chloroflexi	Dehalococcoidia	S085	uncultured_soil_bacterium	uncultured_soil_bacterium	uncultured_soil_bacterium
Modulehubs	Bacteria	Proteobacteria	Alphaproteobacteria	unclassified_Alphaproteobacteria	unclassified_Alphaproteobacteria	unclassified_Alphaproteobacteria	unclassified_Alphaproteobacteria
Modulehubs	Bacteria	Bdellovibrionota	Bdellovibrionia	Bdellovibrionales	Bdellovibrionaceae	Bdellovibrio	unclassified_Bdellovibrio
Modulehubs	Bacteria	Gemmatimonadota	Gemmatimonadetes	Gemmatimonadales	Gemmatimonadaceae	Gemmatimonas	unclassified_Gemmatimonas
Modulehubs	Bacteria	Firmicutes	Clostridia	Lachnospirales	Lachnospiraceae	unclassified_Lachnospiraceae	unclassified_Lachnospiraceae
Modulehubs	Bacteria	Firmicutes	Clostridia	Lachnospirales	Lachnospiraceae	unclassified_Lachnospiraceae	unclassified_Lachnospiraceae
Modulehubs	Bacteria	Patescibacteria	Parcubacteria	Candidatus_Moranbacteria	uncultured_soil_bacterium	uncultured_soil_bacterium	uncultured_soil_bacterium
Modulehubs	Bacteria	Armatimonadota	Fimbriimonadia	Fimbriimonadales	Fimbriimonadaceae	unclassified_Fimbriimonadaceae	unclassified_Fimbriimonadaceae
Modulehubs	Bacteria	Bacteroidota	Bacteroidia	Chitinophagales	Chitinophagaceae	unclassified_Chitinophagaceae	unclassified_Chitinophagaceae
Modulehubs	Bacteria	unclassified_Bacteria	unclassified_Bacteria	unclassified_Bacteria	unclassified_Bacteria	unclassified_Bacteria	unclassified_Bacteria
Modulehubs	Bacteria	Verrucomicrobiota	Verrucomicrobiae	Chthoniobacterales	Chthoniobacteraceae	Chthoniobacter	unclassified_Chthoniobacter
Modulehubs	Bacteria	Chloroflexi	Anaerolineae	Caldilineales	Caldilineaceae	unclassified_Caldilineaceae	unclassified_Caldilineaceae
Modulehub	Bacteria	Proteobacteria	Gammaprote	Burkholderiales	Oxalobacteraceae	unclassified_Oxalobact	unclassified_Oxalobactera

s			obacteria			eraceae	ceae
Modulehubs	Bacteria	Chloroflexi	Anaerolineae	Caldilineales	Caldilineaceae	unclassified_Caldilineaceae	unclassified_Caldilineaceae
Modulehubs	Bacteria	Bacteroidota	Bacteroidia	Cytophagales	Spirosomaceae	Dyadobacter	unclassified_Dyadobacter
Modulehubs	Bacteria	Proteobacteria	Alphaproteobacteria	Sphingomonadales	Sphingomonadaceae	Novosphingobium	unclassified_Novosphingobium
Modulehubs	Bacteria	unclassified_Bacteria	unclassified_Bacteria	unclassified_Bacteria	unclassified_Bacteria	unclassified_Bacteria	unclassified_Bacteria
Modulehubs	Bacteria	Verrucomicrobiota	Verrucomicrobiae	Pedosphaerales	Pedosphaeraceae	unclassified_Pedosphaeraceae	unclassified_Pedosphaeraceae
Modulehubs	Bacteria	Gemmatimonadota	Gemmatimonadetes	Gemmatimonadales	Gemmatimonadaceae	Gemmatimonas	unclassified_Gemmatimonas
Modulehubs	Bacteria	Bacteroidota	Bacteroidia	Bacteroidales	Muribaculaceae	unclassified_Muribaculaceae	unclassified_Muribaculaceae

Table S8 Key bacterial communities at 30 days of maize growth under inoculated AM fungi treatment.

type	Kingdom	Phylum	Class	Order	Family	Genus	Species
Connectors	Bacteria	Chloroflexi	Chloroflexia	Thermomicrobiales	JG30_KF_CM45	uncultured_Chloroflexi_bacterium	uncultured_Chloroflexi_bacterium
Connectors	Bacteria	Bdellovibrionota	Bdellovibrionia	Bdellovibrionales	Bdellovibrionaceae	Bdellovibrio	unclassified_Bdellovibrio
Connectors	Bacteria	Proteobacteria	Alphaproteobacteria	Rhizobiales	Beijerinckiaceae	28_YEA_48	Afipia_genosp._6
Connectors	Bacteria	Actinobacteriota	Actinobacteria	Micrococcales	Micrococcaceae	unclassified_Micrococcaceae	unclassified_Micrococcaceae
Connectors	Bacteria	Actinobacteriota	Actinobacteria	Propionibacteriales	Nocardioidaceae	Nocardioides	unclassified_Nocardioides
Connectors	Bacteria	Bacteroidota	Bacteroidia	Cytophagales	Hymenobacteraceae	Adhaeribacter	unclassified_Adhaeribacter
Connectors	Bacteria	Proteobacteria	Gammaproteobacteria	Pseudomonadales	Moraxellaceae	Cavicella	unclassified_Cavicella
Connectors	Bacteria	Proteobacteria	Alphaproteobacteria	Micavibrionales	unclassified_Micavibrionales	unclassified_Micavibrionales	unclassified_Micavibrionales
Connectors	Bacteria	Proteobacteria	Alphaproteobacteria	Micavibrionales	unclassified_Micavibrionales	unclassified_Micavibrionales	unclassified_Micavibrionales
Connectors	Bacteria	Proteobacteria	Gammaproteobacteria	Burkholderiales	Oxalobacteraceae	unclassified_Oxalobacteraceae	unclassified_Oxalobacteraceae
Connectors	Bacteria	Proteobacteria	Alphaproteobacteria	Caulobacterales	Caulobacteraceae	unclassified_Caulobacteraceae	unclassified_Caulobacteraceae
Connectors	Bacteria	Chloroflexi	Chloroflexia	Thermomicrobiales	JG30_KF_CM45	unclassified_JG30_KF_CM45	unclassified_JG30_KF_CM45
Connectors	Bacteria	Bacteroidota	Bacteroidia	Cytophagales	Microscillaceae	unclassified_Microscillaceae	uncultured_Flexibacteraceae_bacterium
Connectors	Bacteria	Verrucomicr	Chlamydiae	Chlamydiales	cvE6	unclassified_cvE6	unclassified_cvE6

		obiota					
Connectors	Bacteria	Proteobacteria	Alphaproteobacteria	Rhizobiales	Rhizobiaceae	Allorhizobium_Neorhizobium_Pararhizobium_Rhizobium	unclassified_Allorhizobium_Neorhizobium_Pararhizobium_Rhizobium
Connectors	Bacteria	Verrucomicrobiota	Verrucomicrobiae	Opitutales	Opitutaceae	Opitutus	unclassified_Opitutus
Connectors	Bacteria	Gemmatimonadota	unclassified_Gemmatimonadota	unclassified_Gemmatimonadota	unclassified_Gemmatimonadota	unclassified_Gemmatimonadota	unclassified_Gemmatimonadota
Modulehubs	Bacteria	Proteobacteria	Gammaproteobacteria	Salinisphaerales	Solimonadaceae	Polycyclovorans	unclassified_Polycyclovorans
Modulehubs	Bacteria	Proteobacteria	Gammaproteobacteria	Burkholderiales	Nitrosomonadaceae	MND1	unclassified_MND1
Modulehubs	Bacteria	Actinobacteriota	Actinobacteria	Frankiales	Sporichthyaceae	hgcI_clade	unclassified_hgcI_clade
Modulehubs	Bacteria	unclassified_Bacteria	unclassified_Bacteria	unclassified_Bacteria	unclassified_Bacteria	unclassified_Bacteria	unclassified_Bacteria
Modulehubs	Bacteria	Gemmatimonadota	Gemmatimonadetes	Gemmatimonadales	Gemmatimonadaceae	Gemmatimonas	unclassified_Gemmatimonas
Modulehubs	Bacteria	Actinobacteriota	Acidimicrobiia	unclassified_Acidimicrobiia	unclassified_Acidimicrobiia	unclassified_Acidimicrobiia	unclassified_Acidimicrobiia
Modulehubs	Bacteria	Chloroflexi	Anaerolineae	SBR1031	uncultured_Chloroflexi_bacterium	uncultured_Chloroflexi_bacterium	uncultured_Chloroflexi_bacterium
Modulehubs	Bacteria	Proteobacteria	Alphaproteobacteria	Rhizobiales	Stappiaceae	Agaricicola	Agaricicola_taiwanensis
Modulehubs	Bacteria	Patescibacteria	Saccharimonadia	Saccharimonadales	unclassified_Saccharimonadales	unclassified_Saccharimonadales	unclassified_Saccharimonadales
Modulehubs	Bacteria	unclassified_Bacteria	unclassified_Bacteria	unclassified_Bacteria	unclassified_Bacteria	unclassified_Bacteria	unclassified_Bacteria
Modulehub	Bacteria	Bacteroidota	Bacteroidia	Bacteroidales	Muribaculaceae	uncultured_Porphyro	uncultured_Porphyromon

s						monadaceae_bacterium	adaceae_bacterium
Modulehubs	Bacteria	Bacteroidota	Bacteroidia	Bacteroidales	Prevotellaceae	Prevotellaceae_UCG_001	unclassified_Prevotellaceae_UCG_001
Modulehubs	Bacteria	Proteobacteria	Alphaproteobacteria	unclassified_Alphaproteobacteria	unclassified_Alphaproteobacteria	unclassified_Alphaproteobacteria	unclassified_Alphaproteobacteria
Modulehubs	Bacteria	unclassified_Bacteria	unclassified_Bacteria	unclassified_Bacteria	unclassified_Bacteria	unclassified_Bacteria	unclassified_Bacteria
Modulehubs	Bacteria	Proteobacteria	Alphaproteobacteria	Rickettsiales	Rickettsiaceae	unclassified_Rickettsiaceae	unclassified_Rickettsiaceae
Modulehubs	Bacteria	Bacteroidota	Bacteroidia	Sphingobacteriales	FFCH9454	unclassified_FFCH9454	unclassified_FFCH9454
Modulehubs	Bacteria	Actinobacteriota	Actinobacteria	Micrococcales	Microbacteriaceae	unclassified_Microbacteriaceae	unclassified_Microbacteriaceae
Modulehubs	Bacteria	Bdellovibrionota	Bdellovibrionia	Bdellovibrionales	Bdellovibrionaceae	Bdellovibrio	unclassified_Bdellovibrio
Modulehubs	Bacteria	Bacteroidota	Bacteroidia	Cytophagales	Microscillaceae	unclassified_Microscillaceae	unclassified_Microscillaceae
Modulehubs	Bacteria	Proteobacteria	Alphaproteobacteria	Rhodospirillales	Rhodospirillaceae	unclassified_Rhodospirillaceae	unclassified_Rhodospirillaceae
Modulehubs	Bacteria	Proteobacteria	Alphaproteobacteria	Rhizobiales	Devosiaceae	Pelagibacterium	unclassified_Pelagibacterium
Modulehubs	Bacteria	Actinobacteriota	Acidimicrobiia	Microtrichales	unclassified_Microtrichales	unclassified_Microtrichales	unclassified_Microtrichales
Modulehubs	Bacteria	Proteobacteria	Alphaproteobacteria	unclassified_Alphaproteobacteria	unclassified_Alphaproteobacteria	unclassified_Alphaproteobacteria	unclassified_Alphaproteobacteria
Modulehubs	Bacteria	Bdellovibrionota	Oligoflexia	053A03_B_DI_P58	uncultured_delta_proteobacterium	uncultured_delta_proteobacterium	uncultured_delta_proteobacterium
Modulehubs	Bacteria	Proteobacteria	Alphaproteobacteria	Rhizobiales	Hyphomicrobiaceae	Pedomicrobium	unclassified_Pedomicrobium

Table S9 Key bacterial communities at 60 days of maize growth under inoculated AM fungi treatment.

type	Kingdom	Phylum	Class	Order	Family	Genus	Species
Connectors	Bacteria	Patescibacteria	Parcubacteria	unclassified_Parcubacteria	unclassified_Parcubacteria	unclassified_Parcubacteria	unclassified_Parcubacteria
Connectors	Bacteria	Firmicutes	Bacilli	Exiguobacterales	Exiguobacteraceae	Exiguobacterium	unclassified_Exiguobacterium
Connectors	Bacteria	Acidobacteriota	Holophagae	Subgroup_7	uncultured_Acidobacterium_sp.	uncultured_Acidobacterium_sp.	uncultured_Acidobacterium_sp.
Connectors	Bacteria	unclassified_Bacteria	unclassified_Bacteria	unclassified_Bacteria	unclassified_Bacteria	unclassified_Bacteria	unclassified_Bacteria
Connectors	Bacteria	Firmicutes	Clostridia	Oscillospirales	Ruminococcaceae	[Eubacterium]_siraeum_group	unclassified_[Eubacterium]_siraeum_group
Connectors	Bacteria	Firmicutes	Clostridia	Lachnospirales	Lachnospiraceae	unclassified_Lachnospiraceae	unclassified_Lachnospiraceae
Connectors	Bacteria	Proteobacteria	Alphaproteobacteria	Rhizobiales	Beijerinckiaceae	Microvirga	unclassified_Microvirga
Connectors	Bacteria	Patescibacteria	Parcubacteria	Candidatus_Kaiserbacteria	Candidatus_Kaiserbacteria_bacterium_RIFOXYB1_FULL_46_14	unclassified_Candidatus_Kaiserbacteria_bacterium_RIFOXYB1_FULL_46_14	unclassified_Candidatus_Kaiserbacteria_bacterium_RIFOXYB1_FULL_46_14
Connectors	Bacteria	Proteobacteria	Alphaproteobacteria	Acetobacterales	Acetobacteraceae	Siccirubricoccus	Siccirubricoccus_deserti
Connectors	Bacteria	Proteobacteria	Gammaproteobacteria	Burkholderiales	Nitrosomonadaceae	Ellin6067	unclassified_Ellin6067
Connectors	Bacteria	Proteobacteria	Gammaproteobacteria	Burkholderiales	Oxalobacteraceae	unclassified_Oxalobacteraceae	unclassified_Oxalobacteraceae
Connectors	Bacteria	Abditibacteriota	Abditibacteria	Abditibacteriales	Abditibacteriaceae	Abditibacterium	unclassified_Abditibacterium
Connectors	Bacteria	Proteobacteri	Alphaprote	Sphingomonada	Sphingomonadaceae	Altererythrobacter	unclassified_Altererythrob

		a	obacteria	les			acter
Connectors	Bacteria	Proteobacteria	Gammaproteobacteria	Burkholderiales	Methylophilaceae	unclassified_Methylophilaceae	unclassified_Methylophilaceae
Connectors	Bacteria	Proteobacteria	Alphaproteobacteria	Rhizobiales	Xanthobacteraceae	Pseudolabrys	unclassified_Pseudolabrys
Connectors	Bacteria	Proteobacteria	Alphaproteobacteria	Rhizobiales	unclassified_Rhizobiales	unclassified_Rhizobiales	unclassified_Rhizobiales
Connectors	Bacteria	Proteobacteria	Gammaproteobacteria	Diplorickettsiales	Diplorickettsiaceae	unclassified_Diplorickettsiaceae	unclassified_Diplorickettsiaceae
Modulehubs	Bacteria	Proteobacteria	Gammaproteobacteria	unclassified_Gammaproteobacteria	unclassified_Gammaproteobacteria	unclassified_Gammaproteobacteria	unclassified_Gammaproteobacteria
Modulehubs	Bacteria	Proteobacteria	Alphaproteobacteria	unclassified_Alphaproteobacteria	unclassified_Alphaproteobacteria	unclassified_Alphaproteobacteria	unclassified_Alphaproteobacteria
Modulehubs	Bacteria	Gemmatimonadota	unclassified_Gemmatimonadota	unclassified_Gemmatimonadota	unclassified_Gemmatimonadota	unclassified_Gemmatimonadota	unclassified_Gemmatimonadota
Modulehubs	Bacteria	Patescibacteria	Parcubacteria	Candidatus_Kaiserbacteria	unclassified_Candidatus_Kaiserbacteria	unclassified_Candidatus_Kaiserbacteria	unclassified_Candidatus_Kaiserbacteria
Modulehubs	Bacteria	Gemmatimonadota	Longimicrobia	Longimicrobiales	Longimicrobiaceae	unclassified_Longimicrobiaceae	unclassified_Longimicrobiaceae
Modulehubs	Bacteria	Actinobacteriota	Acidimicrobiia	unclassified_Acidimicrobiia	unclassified_Acidimicrobiia	unclassified_Acidimicrobiia	unclassified_Acidimicrobiia
Modulehubs	Bacteria	Elusimicrobiota	Elusimicrobia	Lineage_IV	uncultured_Termite_group_1_bacterium	uncultured_Termite_group_1_bacterium	uncultured_Termite_group_1_bacterium
Modulehubs	Bacteria	Chloroflexi	Anaerolineae	SBR1031	uncultured_Chloroflexi_bacterium	uncultured_Chloroflexi_bacterium	uncultured_Chloroflexi_bacterium
Modulehubs	Bacteria	Actinobacteriota	Thermoleophilia	Gaiellales	unclassified_Gaiellales	unclassified_Gaiellales	unclassified_Gaiellales

Modulehubs	Bacteria	Firmicutes	Clostridia	Lachnospirales	Lachnospiraceae	Roseburia	Roseburia_intestinalis
Modulehubs	Bacteria	Bacteroidota	Bacteroidia	Bacteroidales	Prevotellaceae	Prevotellaceae_UCG_003	unclassified_Prevotellaceae_UCG_003
Modulehubs	Bacteria	Gemmatimonadota	unclassified_Gemmatimonadota	unclassified_Gemmatimonadota	unclassified_Gemmatimonadota	unclassified_Gemmatimonadota	unclassified_Gemmatimonadota
Modulehubs	Bacteria	Myxococcota	Myxococcia	Myxococcales	Myxococcaceae	unclassified_Myxococcaceae	unclassified_Myxococcaceae
Modulehubs	Bacteria	unclassified_Bacteria	unclassified_Bacteria	unclassified_Bacteria	unclassified_Bacteria	unclassified_Bacteria	unclassified_Bacteria
Modulehubs	Bacteria	Chloroflexi	Dehalococcoidia	SAR202_clade	uncultured_Chloroflexi_bacterium	uncultured_Chloroflexi_bacterium	uncultured_Chloroflexi_bacterium
Modulehubs	Bacteria	Bacteroidota	Bacteroidia	Bacteroidales	Muribaculaceae	unclassified_Muribaculaceae	unclassified_Muribaculaceae
Modulehubs	Bacteria	Firmicutes	Clostridia	Lachnospirales	Lachnospiraceae	Roseburia	unclassified_Roseburia
Modulehubs	Bacteria	Bdellovibrionota	Oligoflexia	0319_6G20	uncultured_Anaeromyxobacter_sp.	uncultured_Anaeromyxobacter_sp.	uncultured_Anaeromyxobacter_sp.
Modulehubs	Bacteria	Firmicutes	Bacilli	Paenibacillales	Paenibacillaceae	unclassified_Paenibacillaceae	uncultured_Firmicutes_bacterium
Modulehubs	Bacteria	unclassified_Bacteria	unclassified_Bacteria	unclassified_Bacteria	unclassified_Bacteria	unclassified_Bacteria	unclassified_Bacteria
Modulehubs	Bacteria	Elusimicrobiota	Elusimicrobia	Lineage_IV	uncultured_Termite_group_1_bacterium	uncultured_Termite_group_1_bacterium	uncultured_Termite_group_1_bacterium
Modulehubs	Bacteria	unclassified_Bacteria	unclassified_Bacteria	unclassified_Bacteria	unclassified_Bacteria	unclassified_Bacteria	unclassified_Bacteria
Modulehubs	Bacteria	Chloroflexi	unclassified_Chloroflexi	unclassified_Chloroflexi	unclassified_Chloroflexi	unclassified_Chloroflexi	unclassified_Chloroflexi

Modulehubs	Bacteria	Proteobacteria	Alphaproteobacteria	Rhizobiales	Hyphomicrobiaceae	unclassified_Hyphomicrobiaceae	unclassified_Hyphomicrobiaceae
Modulehubs	Bacteria	Patescibacteria	Parcubacteria	Candidatus_Kaiserbacteria	uncultured_soil_bacterium	uncultured_soil_bacterium	uncultured_soil_bacterium
Modulehubs	Bacteria	Patescibacteria	Saccharimonadia	Saccharimonadales	uncultured_Candidatus_Saccharibacteria_bacterium	uncultured_Candidatus_Saccharibacteria_bacterium	uncultured_Candidatus_Saccharibacteria_bacterium
Modulehubs	Bacteria	Chloroflexi	unclassified_Chloroflexi	unclassified_Chloroflexi	unclassified_Chloroflexi	unclassified_Chloroflexi	unclassified_Chloroflexi
Modulehubs	Bacteria	Proteobacteria	Alphaproteobacteria	Acetobacterales	Acetobacteraceae	Roseomonas	unclassified_Roseomonas
Modulehubs	Bacteria	Bacteroidota	Rhodothermia	Rhodothermales	Rhodothermaceae	unclassified_Rhodothermaceae	unclassified_Rhodothermaceae
Modulehubs	Bacteria	Chloroflexi	unclassified_Chloroflexi	unclassified_Chloroflexi	unclassified_Chloroflexi	unclassified_Chloroflexi	unclassified_Chloroflexi
Modulehubs	Bacteria	Gemmatimonadota	Longimicrobia	Longimicrobiales	Longimicrobiaceae	unclassified_Longimicrobiaceae	unclassified_Longimicrobiaceae
Modulehubs	Bacteria	Proteobacteria	Gammaproteobacteria	Burkholderiales	Alcaligenaceae	Achromobacter	unclassified_Achromobacter
Modulehubs	Bacteria	Bacteroidota	Bacteroidia	Bacteroidales	Muribaculaceae	unclassified_Muribaculaceae	unclassified_Muribaculaceae
Modulehubs	Bacteria	Firmicutes	Bacilli	Lactobacillales	Streptococcaceae	Lactococcus	unclassified_Lactococcus
Modulehubs	Bacteria	Bacteroidota	Bacteroidia	Chitinophagales	unclassified_Chitinophagales	unclassified_Chitinophagales	unclassified_Chitinophagales
Modulehubs	Bacteria	Bacteroidota	Bacteroidia	Bacteroidales	Muribaculaceae	uncultured_Bacteroidales_bacterium	uncultured_Bacteroidales_bacterium

Table S10 Key bacterial communities at 90 days of maize growth under inoculated AM fungi treatment.

type	Kingdom	Phylum	Class	Order	Family	Genus	Species
Connectors	Bacteria	Firmicutes	Bacilli	Alicyclobacillales	Alicyclobacillaceae	Tumebacillus	unclassified_Tumebacillus
Connectors	Bacteria	Actinobacteriota	Actinobacteria	Propionibacteriales	Nocardioidaceae	Nocardioides	unclassified_Nocardioides
Connectors	Bacteria	Proteobacteria	Gammaproteobacteria	Enterobacterales	Erwiniaceae	Rosenbergiella	unclassified_Rosenbergiella
Connectors	Bacteria	Patescibacteria	Saccharimonadia	Saccharimonadales	unclassified_Saccharimonadales	unclassified_Saccharimonadales	unclassified_Saccharimonadales
Connectors	Bacteria	Thermotogota	Thermotogae	Petrotogales	Petrotogaceae	Defluviitoga	unclassified_Defluviitoga
Connectors	Bacteria	Proteobacteria	Gammaproteobacteria	Burkholderiales	Comamonadaceae	Ramlibacter	unclassified_Ramlibacter
Connectors	Bacteria	Proteobacteria	Alphaproteobacteria	Caulobacterales	Caulobacteraceae	unclassified_Caulobacteraceae	unclassified_Caulobacteraceae
Connectors	Bacteria	unclassified_Bacteria	unclassified_Bacteria	unclassified_Bacteria	unclassified_Bacteria	unclassified_Bacteria	unclassified_Bacteria
Connectors	Bacteria	Bacteroidota	Bacteroidia	Bacteroidales	Muribaculaceae	unclassified_Muribaculaceae	unclassified_Muribaculaceae
Connectors	Bacteria	Bacteroidota	Bacteroidia	Chitinophagales	Chitinophagaceae	Pseudoflavitalea	unclassified_Pseudoflavitalea
Connectors	Bacteria	Proteobacteria	Alphaproteobacteria	Rhizobiales	Devosiaceae	Devosia	unclassified_Devosia
Connectors	Bacteria	Abditibacteriota	Abditibacteria	Abditibacteriales	Abditibacteriaceae	Abditibacterium	unclassified_Abditibacterium
Connectors	Bacteria	Firmicutes	Bacilli	Bacillales	Bacillaceae	Bacillus	Limosilactobacillus_vaginalis
Connectors	Bacteria	Gemmatim	unclassified_	unclassified_	unclassified_Gemmati	unclassified_Gemmatimo	unclassified_Gemmatimon

		onadota	Gemmatimo nadota	Gemmatimo nadota	monadota	nadota	adota
Connectors	Bacteria	Gemmatim onadota	unclassified_ Gemmatimo nadota	unclassified_ Gemmatimo nadota	unclassified_Gemmati monadota	unclassified_Gemmatimo nadota	unclassified_Gemmatimon adota
Modulehub s	Bacteria	Patescibact eria	Parcubacteri a	unclassified_ Parcubacteri a	unclassified_Parcubact eria	unclassified_Parcubacteri a	unclassified_Parcubacteria
Modulehub s	Bacteria	Bacteroidot a	Bacteroidia	Cytophagale s	Spirosomaceae	Larkinella	unclassified_Larkinella
Modulehub s	Bacteria	Gemmatim onadota	Gemmatimo nadetes	Gemmatimo nadales	Gemmatimonadaceae	unclassified_Gemmatimo nadaceae	unclassified_Gemmatimon adaceae
Modulehub s	Bacteria	Proteobacte ria	Gammaprote obacteria	Diplorickett siales	Diplorickettsiaceae	unclassified_Diplorickett siaceae	uncultured_Coxiellaceae_ bacterium
Modulehub s	Bacteria	Bdellovibri onota	Oligoflexia	053A03_B_DI _P58	uncultured_delta_prot eobacterium	uncultured_delta_proteob acterium	uncultured_delta_proteob acterium
Modulehub s	Bacteria	Bacteroidot a	Bacteroidia	Chitinophag ales	Chitinophagaceae	Flavitalea	unclassified_Flavitalea
Modulehub s	Bacteria	Patescibact eria	Parcubacteri a	Candidatus_ Doudnabacte ria	Candidatus_Doudnab acteria_bacterium_RIF CSPLOWO2_02_FULL _48_13	unclassified_Candidatus_ Doudnabacteria_bacteriu m_RIFCSPLOWO2_02_F ULL_48_13	unclassified_Candidatus_ Doudnabacteria_bacteriu m_RIFCSPLOWO2_02_FU LL_48_13
Modulehub s	Bacteria	Proteobacte ria	Alphaproteo bacteria	unclassified_ Alphaproteo bacteria	unclassified_Alphapro teobacteria	unclassified_Alphaproteo bacteria	unclassified_Alphaproteob acteria
Modulehub s	Bacteria	Fibrobacter ota	Fibrobacteria	Fibrobacteral es	Fibrobacteraceae	possible_genus_04	unclassified_possible_gen us_04
Modulehub s	Bacteria	Elusimicro biota	Elusimicrobi a	MVP_88	unclassified_MVP_88	unclassified_MVP_88	unclassified_MVP_88
Modulehub	Bacteria	Patescibact	Saccharimon	Saccharimon	LWQ8	unclassified_LWQ8	unclassified_LWQ8

s		eria	adia	adales			
Modulehubs	Bacteria	Proteobacteria	Gammaproteobacteria	Burkholderiales	Oxalobacteraceae	Massilia	unclassified_Massilia
Modulehubs	Bacteria	Bdellovibrionota	Bdellovibrionia	Bdellovibrionales	Bdellovibrionaceae	Bdellovibrio	unclassified_Bdellovibrio
Modulehubs	Bacteria	Proteobacteria	Gammaproteobacteria	Diplorickettsiales	Diplorickettsiaceae	unclassified_Diplorickettsiaceae	unclassified_Diplorickettsiaceae
Modulehubs	Bacteria	Proteobacteria	Gammaproteobacteria	Diplorickettsiales	Diplorickettsiaceae	unclassified_Diplorickettsiaceae	unclassified_Diplorickettsiaceae
Modulehubs	Bacteria	Patescibacteria	Saccharimonadia	Saccharimonadales	Saccharimonadaceae	TM7a	unclassified_TM7a
Modulehubs	Bacteria	Proteobacteria	Gammaproteobacteria	unclassified_Gammaproteobacteria	unclassified_Gammaproteobacteria	unclassified_Gammaproteobacteria	unclassified_Gammaproteobacteria
Modulehubs	Bacteria	Proteobacteria	Gammaproteobacteria	Burkholderiales	TRA3_20	uncultured_gamma_proteobacterium	uncultured_gamma_proteobacterium
Modulehubs	Bacteria	Proteobacteria	Alphaproteobacteria	Rickettsiales	SM2D12	unclassified_SM2D12	unclassified_SM2D12
Modulehubs	Bacteria	Planctomycetota	Planctomycetes	Pirellulales	Pirellulaceae	Pirellula	unclassified_Pirellula
Modulehubs	Bacteria	Chloroflexi	Chloroflexia	Thermomicrobiales	AKYG1722	unclassified_AKYG1722	unclassified_AKYG1722
Modulehubs	Bacteria	Proteobacteria	Gammaproteobacteria	Enterobacterales	Pasteurellaceae	Rodentibacter	unclassified_Rodentibacter
Modulehubs	Bacteria	unclassified_Bacteria	unclassified_Bacteria	unclassified_Bacteria	unclassified_Bacteria	unclassified_Bacteria	unclassified_Bacteria
Modulehubs	Bacteria	Chloroflexi	Chloroflexia	Thermomicrobiales	JG30_KF_CM45	unclassified_JG30_KF_CM45	unclassified_JG30_KF_CM45
Modulehubs	Bacteria	Acidobacteriota	Acidobacteriae	Bryobacterales	Bryobacteraceae	Bryobacter	unclassified_Bryobacter

Modulehubs	Bacteria	Proteobacteria	Alphaproteobacteria	Reyranellales	Reyranellaceae	unclassified_Reyranellaceae	uncultured_Alphaproteobacteria_bacterium
Modulehubs	Bacteria	Gemmatimonadota	unclassified_Gemmatimonadota	unclassified_Gemmatimonadota	unclassified_Gemmatimonadota	unclassified_Gemmatimonadota	unclassified_Gemmatimonadota
Modulehubs	Bacteria	Bdellovibrionota	Bdellovibrionia	Bdellovibrionales	Bdellovibrionaceae	Bdellovibrio	unclassified_Bdellovibrio
Modulehubs	Bacteria	Proteobacteria	Gammaproteobacteria	Diplorickettsiales	Diplorickettsiaceae	unclassified_Diplorickettsiaceae	unclassified_Diplorickettsiaceae
Modulehubs	Bacteria	Proteobacteria	Gammaproteobacteria	Acidiferrobacterales	Acidiferrobacteraceae	Sulfurifustis	unclassified_Sulfurifustis
Modulehubs	Bacteria	Proteobacteria	Gammaproteobacteria	Burkholderiales	Comamonadaceae	Pelomonas	unclassified_Pelomonas
Modulehubs	Bacteria	Bacteroidota	Bacteroidia	Bacteroidales	Prevotellaceae	unclassified_Prevotellaceae	unclassified_Prevotellaceae
Modulehubs	Bacteria	Bacteroidota	Bacteroidia	Chitinophagales	Saprospiraceae	unclassified_Saprospiraceae	uncultured_Saprospiraceae_bacterium
Modulehubs	Bacteria	Firmicutes	Clostridia	Lachnospirales	Lachnospiraceae	unclassified_Lachnospiraceae	unclassified_Lachnospiraceae
Modulehubs	Bacteria	Actinobacteriota	Actinobacteria	Propionibacteriales	Nocardioidaceae	Nocardioides	unclassified_Nocardioides
Modulehubs	Bacteria	Verrucomicrobiota	Verrucomicrobiae	Pedosphaerales	Pedosphaeraceae	unclassified_Pedosphaeraceae	unclassified_Pedosphaeraceae
Modulehubs	Bacteria	Acidobacteriota	Acidobacteriae	Bryobacterales	Bryobacteraceae	Bryobacter	unclassified_Bryobacter
Modulehubs	Bacteria	Chloroflexi	Chloroflexia	Chloroflexales	Roseiflexaceae	unclassified_Roseiflexaceae	unclassified_Roseiflexaceae
Modulehubs	Bacteria	Bacteroidota	Bacteroidia	Bacteroidales	Prevotellaceae	Prevotella	Prevotella_sp._oral_taxon_317_str._F0108
Modulehub	Bacteria	unclassified	unclassified_	unclassified_	unclassified_Bacteria	unclassified_Bacteria	unclassified_Bacteria

s		_Bacteria	Bacteria	Bacteria			
Modulehub s	Bacteria	Proteobacte ria	Alphaproteo bacteria	Kiloniellales	Fodinicurvataceae	unclassified_Fodinicurvat aceae	unclassified_Fodinicurvata ceae
Modulehub s	Bacteria	Proteobacte ria	Gammaprote obacteria	Burkholderia les	Sutterellaceae	Parasutterella	unclassified_Parasutterella
Modulehub s	Bacteria	Proteobacte ria	Alphaproteo bacteria	Rhizobiales	Devosiaceae	Devosia	unclassified_Devosia
